# Heptanuclear Silver Hydride Clusters as Catalytic Precursors for the Reduction of 4-Nitrophenol

**DOI:** 10.3390/molecules27165223

**Published:** 2022-08-16

**Authors:** Tunde L. Yusuf, Segun A. Ogundare, Michael N. Pillay, Werner E. van Zyl

**Affiliations:** 1 School of Chemistry and Physics, Westville Campus, University of KwaZulu-Natal, Chiltern Hills, Durban 4000, South Africa; 2 Department of Chemical Sciences, Olabisi Onabanjo University, Ago-Iwoye 120107, Nigeria

**Keywords:** silver, cluster, hydride, catalysis, dithiophosphonate, complexes, nanoparticles

## Abstract

We report on the design, synthesis, and characterization of the first silver hydride clusters solely protected and stabilized by dithiophosphonate ligands and their application for the in situ generation of silver nanoparticles towards the catalytic reduction of 4-nitrophenol in an aqueous system. The synthesis of the silver monohydride cluster involves the incorporation of an interstitial hydride using sodium borohydride. Poly-nuclear magnetic resonance and mass spectrometry were used to establish the structural properties. The structural properties were then confirmed with a single-crystal X-ray diffraction analysis, which showed a distorted tetracapped tetrahedron core with one hydride ion encapsulated within the core of the silver framework. Additionally, the synthesized heptanuclear silver hydride was utilized as a precursor for the in situ generation of silver nanoparticles, which simultaneously catalyzed the reduction of 4-nitrophenol. The mechanism of the catalytic activity was investigated by first synthesizing AgNPs, which was subsequently used as a catalyst. The kinetic study showed that the pseudo-first constant obtained using the cluster (2.43 × 10^−2^ s^−1^) was higher than that obtained using the synthesized AgNPs (2.43 × 10^−2^ s^−1^). This indicated that the silver monohydride cluster was more active owing to the release of the encapsulated hydride ion and greater reaction surface prior to aggregation.

## 1. Introduction

Since the synthesis of the first binary transition metal hydride reported by Wurtz in 1844 [1], transition metal hydrides have been topical research avenues owing to their interesting structural and bonding properties as well as their application in hydrogen storage technology and catalysis [2,3,4,5,6,7,8,9,10,11,12]. Metal hydrides have been proposed to be a key intermediate in many catalytic processes [13,14,15,16]. For example, the dehydrogenation of alcohol to a ketone catalyzed by silver clusters supported on Al_2_O_3_ is believed to proceed via Ag–H intermediates [17,18]. Silver clusters supported on alumina have also been applied as a heterogeneous catalyst in the direct *N*-alkylation of anilines with benzyl alcohol [19], selective reduction of NO_x_ for automobile application [20,21,22], oxidant free C–H reduction of alcohol, C–C cross-coupling of secondary alcohols with primary alcohols [17,18] and amide synthesis from direct addition of alcohol with silver hydride proposed to be a key intermediate in the catalytic systems. Despite these remarkable and unique properties of transition metal hydrides, the structural aspect of this chemistry has often been hindered by instability and difficulty in isolating suitable crystals for X-ray diffraction studies. A remarkable number of copper hydride clusters of high nuclearity have been synthesized via a ligand-protected route, including Cu_8_(H), Cu_20_(H)_11_ [23,24], Cu_28_(H)_15_ [25], and Cu_32_(H)_20_ [26,27]. The synthesis is achieved via the chemical addition of ligand and copper salt in the presence of a hydride source such as borohydride (BH_4_^−^).

There is, however, a paucity of information available on these metal hydride clusters due to their instability in air. Although mixed silver-transition metal clusters such as [Ru–H–Ag] [28], [Mo–H–Ag] [29], [Mn–H–Ag] [30], [Ir–H–Ag] [31], [Pt–H–Ag] [32] are well known, the ligand protected synthetic method has yielded stable and structurally characterized silver cluster such as Ag_6_(H)_4_ [33], Ag_7_(H) [34] and Ag_8_(H). As ligands, dichalcogenides have become an important class for stabilizing metal clusters. Liu and co-workers have reported the synthesis of Ag clusters capped by dithiophosphate and dithiocarbamate ligands [34]. To date, there is no report of dithiophosphonate solely capped large clusters, presumably owing to their steric demand and asymmetrical nature of the ligand, preventing efficient crystal packing. Rothenberger and co-workers reported the synthesis of Ag_28_ stabilized by dithiophosphonate but employed triphenylphosphine as a secondary ligand to aid the crystallization of silver clusters capped by dithiophosphonates [35].

There is, however, still a dearth of information to be unraveled on the synthesis, structural characterization, and catalytic properties of silver hydride clusters solely capped by dithiophosphonate ligands. In continuing our work on metal complexes and clusters of dithiophosphonates [36,37,38,39], we report the synthesis of four new Ag_7_H clusters protected by dithiophosphonate ligands and demonstrate their application as precursors for in situ synthesis of silver nanoparticles as catalysts in the chemical reduction of 4-nitrophenol (4-NP) to 4-aminophenol (4-AP).

The reduction of 4-NP to 4-AP by borohydride is considered a reliable reaction model for assessing the catalytic properties of nanostructures [40,41]. The US Environmental Protection Agency (EPA) has listed 4-nitrophenol (4-NP) as one of the 129 organic chemicals which are carcinogenic and harmful to humans, animals, and plants due to their persistence, stability and resistance to biodegradation [42]. 4-NP is abundant in industrial effluents and soil because of its importance in producing dye, pharmaceuticals, herbicides, and insecticides [42,43,44]. Therefore, the reduction of 4-NP to 4-AP is a more sustainable solution since 4-AP is environmentally benign and an essential precursor in many organic syntheses.

Noble metal nanoparticles have been extensively researched as catalysts for the reduction of 4-NP [45], and there are several reports on AgNPs as an efficient catalyst for the reduction of 4-NP to 4-AP [46,47]. Still, there are only a few reports of silver clusters as a catalyst for this reduction reaction. This is the first structurally characterized silver hydride cluster utilized as a catalyst in the reduction of 4-NP to 4-AP.

## 2. Results and Discussion

### 2.1. Synthesis of the Silver Clusters

**Scheme 1:**
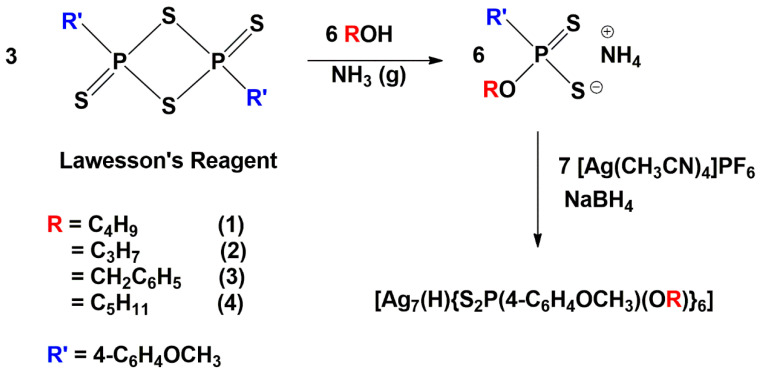
The reaction scheme for the synthesis of the silver monohydride clusters.

Clusters **1**–**4** were synthesized in good yield (>70%) from the reaction between [Ag(CH_3_CN)_4_]PF_6_, dithiophosphonate (DTP) ligand, and sodium borohydride in a ratio of 7:6:1 under a nitrogen atmosphere for 3 h in THF (Scheme 1). The DTP ligands were derivatized using various alcohol; we observed that clusters from MeOH, EtOH, were unstable at room temperature and could not be characterized further. The clusters were isolated as a yellow powder, soluble in toluene, DCM, chloroform, acetone, and benzene and insoluble in hexane, water, and alcohol. All clusters were characterized by elemental analysis, electrospray ionization mass spectrometry, multinuclear NMR and UV-Visible spectroscopy. The structure of **1** was determined by single-crystal X-ray diffraction, whilst data was also collected for **3** and **4**, but unfortunately was of poor quality (see Supporting Information). The synthesis of the silver cluster includes the use of sodium borohydride, allowing for the possibility of an interstitial hydride encapsulated within the cluster. To explore this, the synthesis of **1** and **2** were repeated using NaBD_4_ instead of NaBH_4_ as a hydride source; (**1_D_** and **2_D_**) and investigated using the ^1^H-NMR and high-resolution mass spectrometry. The hydride provided by BH_4_^−^ acts as the anionic template to yield [Ag_7_(H)L_6_].

### 2.2. Nuclear Magnetic Resonance (NMR) Spectroscopy

The ^1^H-NMR for clusters **1**–**4** showed all the ligand functionalities. An additional quartet peak was observed in the spectra of **1** and **2** at 4.11 and 3.77 ppm (Figure S1a,b, Supplementary Materials), respectively, which integrates as 1 relative to the 18 methoxy protons on the anisole ring of the six capping DTP ligands which suggested the presence of an interstitial hydride within the cluster. To further confirm this, the synthesis of **1** and **2** were repeated using deuterated sodium borohydride (NaBD_4_) as a hydride source instead of NaBH_4_; **1_D_** and **2_D_**. The disappearance of the peaks observed at 4.11 and 3.43 ppm confirmed the presence of an interstitial hydride within the heptanuclear core. The ^1^H-NMR of **3** and **4** also displayed all the ligand functionalities and additional broad peaks at 4.71 and 4.01 ppm, respectively, and integrated to 1 relative to 18 methoxy protons of the six anisole rings. Only one singlet peak was observed for all clusters in the solution ^31^P-NMR spectra, which appeared at 108, 107, 101 and 103 ppm for **1**, **2**, **3** and **4**, respectively. Possible peaks that could be associated with precursor PF_6_^−^ counter anion was completely absent, as observed in the ^31^P-NMR spectra of the clusters (Figures S2 and S3, Supplementary Materials). The singlet peak found in the ^31^P-NMR spectra suggested that all the phosphorus atoms were in a chemically and magnetically equivalent environment. The possibility of an encapsulated hydride in an M_7_(H)L_6_ and M_8_(H)L_6_ (L = dithiocarbamate or dithiophosphate) has been proven unequivocally by Liu and co-workers by neutron diffraction analysis in Cu_7_(H) and NMR (^2^H-NMR, ^109^Ag) [48]. In the M_7_ systems, the hydride (H^−^) is required to balance the charge in an otherwise stable and overall neutral cluster containing seven mono-cation metals and six mono-anionic ligands. This work employed the use of unsymmetrical dithiophosphonate ligands (allowing for the potential formation of isomers) instead of symmetrical dithiocarbamate and dithiophosphate used by Liu and co-workers.

### 2.3. Mass Spectrometry

The chemical composition of **1**–**4** was further confirmed by positive ion electrospray ionization mass spectrometry (ESI-MS). The molecular ion peaks for clusters **1** and **1_D_** expected at 2408.17 and 2409.18, respectively, were not observed. However, the HRMS spectra (Figures S4 and S5, Supplementary Materials) of clusters **1** and **1_D_** showed adduct peaks at m/z: 2516.425 (calcd 2516.037) and 2517.447 (calcd 2517.043), which were equivalent to the entire molecular species with an additional silver ion to form an adduct ion corresponding to a mono-charged ion of [Ag_7_(H)L_6_ + (Ag)]^+^ and [Ag_7_(D)L_6_ + (Ag)]^+^, respectively. This is consistent with previously reported Ag_7_(H)L_6_ [34] and Cu_7_(H)(dtc)_6_ (dtc = dithiocarbamate) [48] clusters, which indicates that the neutral Ag_7_ clusters bind strongly with Ag^+^ under ESI gas-phase conditions. Positive mode ESI-MS was also used to determine the chemical composition of **2**. The peak at *m*/*z*: 2432.30 in the HRMS spectrum (Figure S6, Supplementary Materials) of cluster **2** corresponded to [Ag_7_(H)L_6_ + (Ag)]^+^. Similarly, its low-resolution mass spectrum (Figure S7, Supplementary Materials) also displayed fragments at *m*/*z*: 2322.40, 1952.50, 1584.58, 1214.85, and 846.73 corresponding to Ag_7_(H)L_6_, Ag_6_(H)L_5_, Ag_5_(H)L_4_, Ag_4_(H)L_3_, Ag_3_(H)L_2_, respectively. The HRMS spectrum (Figure S8, Supplementary Materials) of cluster **4** also showed a molecular ion peak at 2600.53 (calcd 2600.20) corresponding to [Ag_7_(H)L_6_ + Ag]^+^. However, for cluster **3**, the molecular ion peak expected at 2612.26 was not observed. Instead, an M^2+^ peak at 1468.00 (calcd 1468.00) corresponding to [Ag_7_(H)L_6_ + Ag]^2+^ was observed in the mass spectrum (Figure S9, Supplementary Materials).

### 2.4. X-ray Crystallography

**Figure 1:**
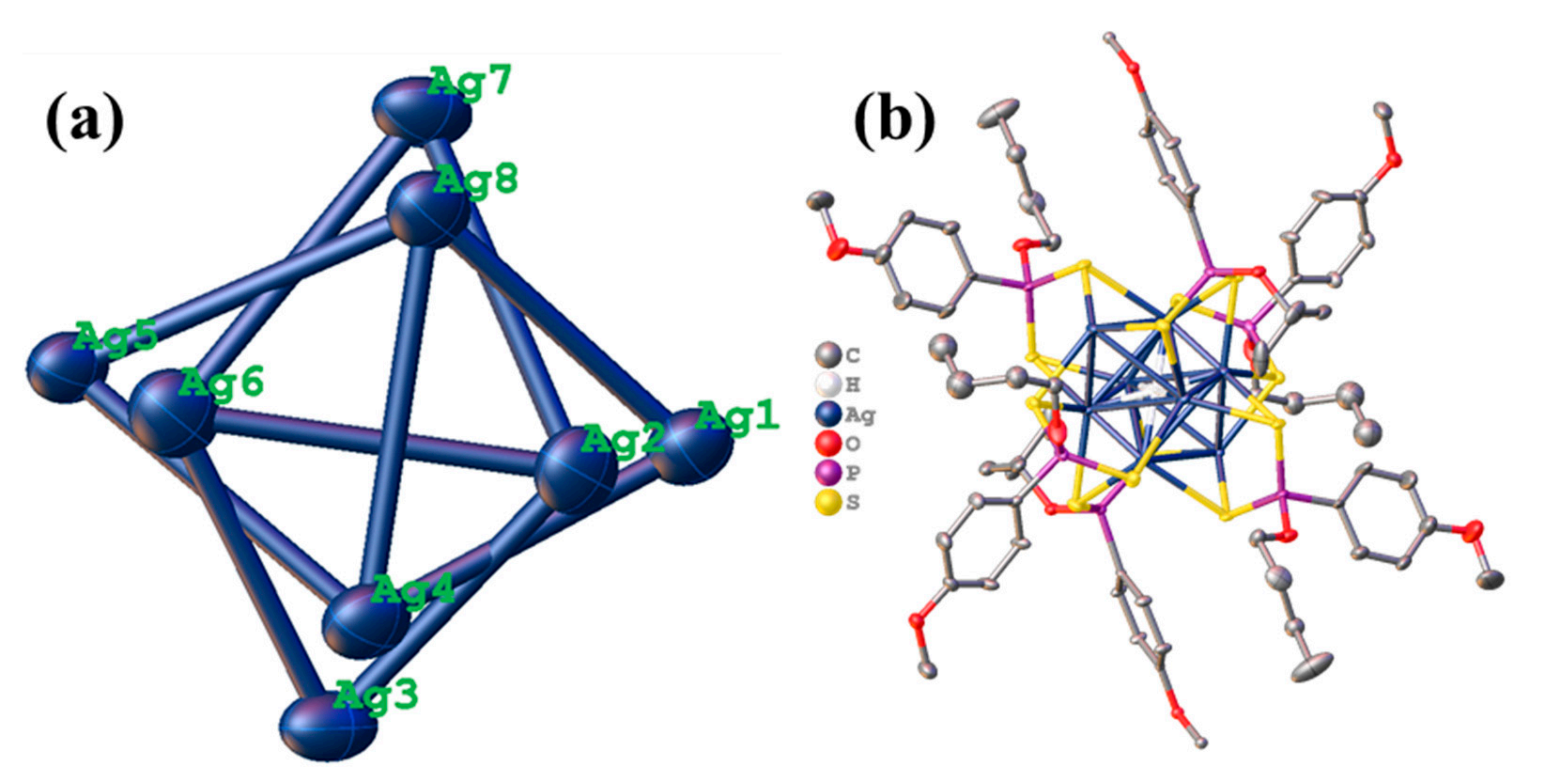

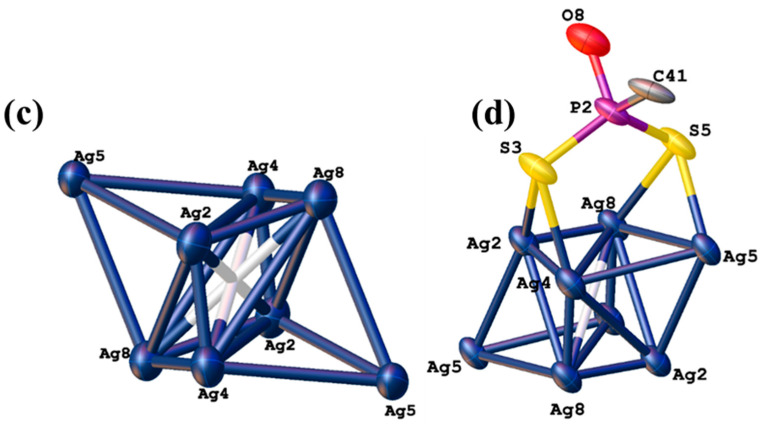
(**a**) Molecular structure of cluster **1**. H-atoms omitted for clarity except for the interstitial hydride. (**b**) Molecular representation of the asymmetric unit for **1** showing only the silver atoms. (**c**) Ag_7_H core structural representation. (**d**) Coordination motif of DTP ligand to the silver core.

Cluster **1**—The crystal structure of **1** revealed that the heptanuclear neutral silver cluster crystalized in a triclinic P (−1) space group with the seven silver atoms disordered in sixteen positions. The asymmetric unit showed two distorted tetrahedrons disordered over each other, as shown in Figure 1. The entire silver framework formed a cage with an encapsulated hydride within the heptanuclear cage. The occupancy for each of the silver atoms in one of the tetrahedrons in the asymmetric unit was fixed at 50% (0.5 × 8 = 4), accounting for four of the silver atoms in the molecule. To account for the remaining three silver atoms in eight positions, two Ag units were fixed at 50%, two at 43%, two at 44%, and two at 13%. The silver framework adopts a distorted tetracapped tetrahedron core with one hydride ion encapsulated within the core. The Ag_7_ core is surrounded by 12 S atoms from 6 bridging dithiophosphonate ligands in a distorted icosahedral cage. A similar disorder is observed on related dithio-based ligands in Ag_7_ [35], Cu_7_ [48], Ag_8_ [49] clusters. The vertex silver atoms in the inner tetrahedron consisted of Ag_1_, Ag_3_ Ag_5_ and Ag_7_ (abbreviated as Ag_cap_) and capped by Ag_2_, Ag_4_, Ag_8_, and Ag_6_ (abbreviated as Ag_v_). The distances Ag_v_–Ag_v_ fall in the range 3.0522(17)–3.2201(17) Å, while those of Ag_v_–Ag_cap_ fall in the range 2.9763(19)–3.3019(18) Å. The dithiophosphonate ligands are coordinated in a tetraconnective tetrametallic (µ_2_, µ_2_) mode. This presents the first report of dithiophosphonate ligand coordinated to silver atoms in this coordination pattern (µ_2_, µ_2_). For the Ag–S distances, the Ag_v_–S ranges from 2.564–2.739Å, and the Ag_cap_–S distances are within the range of 2.342(3)–2.564(3) Å. The distances for Ag_v_–S are longer than those of Ag_cap_–S, which are normal and well within the expected range. The interstitial hydride is located on a crystallographic center of inversion. The distances between the encapsulated hydride and the silver atoms are in the range of 1.987–2.061 Å. The Ag–H distances are comparable to those of Ag_7_ and Ag_8_ reported [34,49,50]. Furthermore, four anisole rings of the six ligands are disordered over two positions and are fixed at 50% occupancy each.

## 3. In Situ Production of Silver Nanoparticle and Reduction of 4-Nitrophenol

**Scheme 2:**
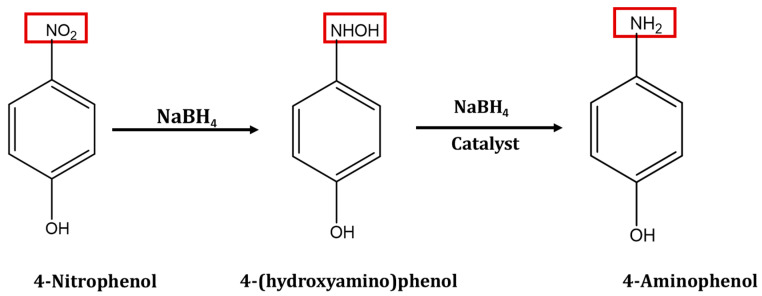
Catalytic reduction of 4-NP to 4-AP.

**Figure 2:**
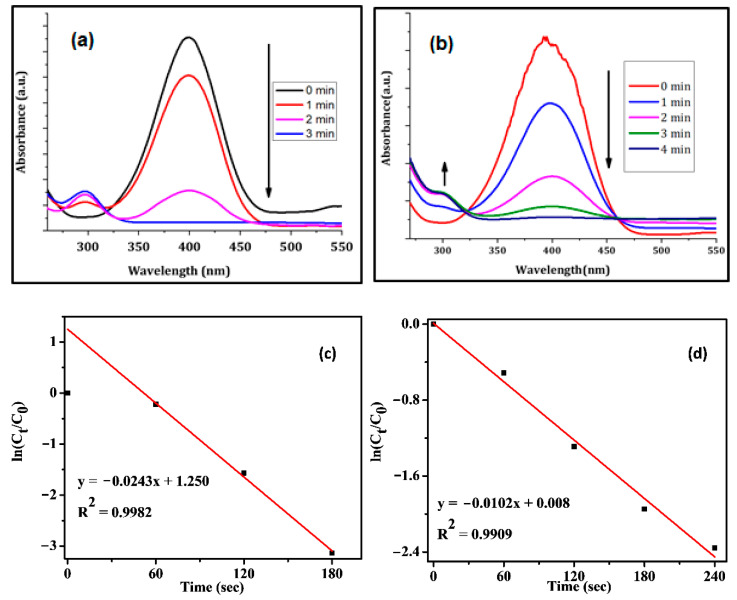
UV-vis spectra showing the successive reduction of 4-NP to 4-AP in the presence of (**a**) cluster **1** (corresponding kinetic plots (**c**)) and (**b**) the derived AgNPs at room temperature (corresponding kinetic plots (**d**)).

Silver nanoparticles have been reported to show outstanding catalytic activity and selectivity in the reduction of 4-NP [51]. The catalytic efficiency of the Ag_7_ cluster was examined via the reduction of 4-NP to 4-AP. To access the role of the interstitial hydride within the Ag_7_ cluster, the catalytic properties of the silver precursor [Ag(CH_3_CN)_4_]PF_6_ were also investigated. The reaction was carried out in a one-step method, and the change in absorbance was monitored over time using UV-visible spectroscopy at one-minute intervals. Cluster **1** was added to a mixture of 4-NP and NaBH_4_ in a 4 mL cuvette. The catalytic property of the cluster was examined by monitoring the spectral changes in the absorption spectrum of 4-(hydroxyamino)phenol. The spectroscopic monitoring showed a rapid reduction of 4-NP. It was established that the reduction reaction of 4-NP to 4-AP proceeds through an intermediate 4-(hydroxyamino)phenol (Scheme 2). Upon the addition of NaBH_4_ to 4-NP, the band of 4-NP immediately shifted from 317 nm to 400 nm, which indicated the formation of 4-nitrophenolate, which has been established as the first stable intermediate form. In the absence of a catalyst, the absorbance of the intermediate remained unchanged after several hours, which suggested that the reaction did not proceed in the absence of a catalyst. The procedure was repeated with the addition of precursor [Ag(CH_3_CN)_4_]PF_6_ as a catalyst, but no significant change was observed in the absorption maxima after several hours, which suggested that the silver starting material does not catalyze the reduction of 4-NP. However, when cluster **1** was introduced as a catalyst, the band at 400 nm started to decrease with time, and a corresponding new band appeared at 300 nm, which indicated the formation 4-AP (Figure 2a). It was observed that a simultaneous reduction of silver ions in cluster **1** used as a catalyst occurred alongside the reduction of 4-NP, which indicated that the catalytic activity of cluster **1** proceeded via the in situ generation of AgNPs. This was further investigated by first chemically reducing cluster **1** using NaBH_4_; the AgNPs isolated were characterized and subsequently used as a catalyst following a similar procedure. The result of the study showed that the derived AgNPs from cluster **1** were equally active as a catalyst for the complete reduction of 4-NP.

When cluster **1** was used as a catalyst, the catalytic reduction of 4-NP was driven to completion in about 3 min, while with AgNPs, the reduction of the reaction of 4-NP took about 4 min. The observed difference in time of completion indicated that the in situ generation of AgNPs (as opposed to adding previously isolated AgNPs) provided improved catalytic efficiency, which suggests greater activity resulting from the higher surface area of the in situ generated AgNPs prior to aggregation and also the release of the active participation of the interstitial hydride ion within cluster **1**. Since excess NaBH_4_ was used in comparison with 4-NP, the reaction is proposed to be a pseudo-first-order reaction. The kinetics of the reaction may be described as ln(C/C_o_) = −kt, where k is the first-order rate constant (min^−1^), t is reaction time; C is the concentration of 4-NP at time t, and C_o_ is a concentration of 4-NP at time 0. The rate constant k of cluster **1** was calculated to be 2.43 × 10^−2^ s^−1^ with a correlation coefficient of 0.9982, while for the derived AgNPs, the rate constant k was calculated to be 1.02 × 10^−2^ s^−1^ with a correlation coefficient of 0.9909. It has been previously shown that the presence of highly reactive silver hydride Ag–H, within the cluster and in the presence of NaBH_4_ is considered a key mediator to the catalytic performance of the silver clusters for the reduction of 4-NP by aiding the transfer of hydrogen in the system [52].

Under similar conditions, the catalytic properties of cluster **1** and AgNPs are comparable with the reaction time of 3 min and 4 min, respectively, and corresponding percentage reduction of 96% and 92%, respectively. Cluster **1** transformed to AgNPs after the first cycle. This prompted the investigation and characterization of the AgNPs, which revealed that the AgNPs were generated in situ and remained stable and reusable after recovery and washing with water via centrifugation. The percentage reduction was ~90% after 5 cycles (Figure S12, Supplementary Materials). The catalytic activity of silver nanoparticles is attributed to the presence of readily available surface electrons on the nanoparticles. The activity of cluster **1** is due to the presence of very reactive silver hydride, and the subsequent rich electrons available at the surface of the in situ generated AgNPs.

## 4. Properties of Cluster 1 and the Synthesized Silver Nanoparticles

The properties were elucidated using high-resolution transmission electron microscopy (HRTEM), scanning electron microscopy (SEM), Fourier-transform infrared (FT-IR) and UV-visible (UV-vis) spectroscopies.

**Figure 3:**
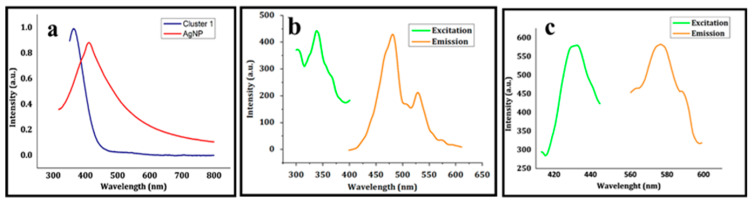
(**a**) Electronic absorption spectrum of cluster **1** (Blue) and AgNPs (red). (**b**) Excitation spectrum of AgNPs (green) and emission spectrum (orange). (**c**) Excitation spectrum of cluster **1** (green) and emission spectrum (orange).

The electronic spectra of cluster **1** and the AgNPs recorded in dichloromethane and water, respectively, showed peaks at 340 nm (cluster **1**) and 420 nm (AgNPs) (Figure 3a). The absorption peak of the AgNPs indicated the localized surface plasmon resonance characteristics of AgNPs. Unlike the synthesized AgNPs, cluster **1** showed absorption maxima at a shorter wavelength in the ultraviolet region, which suggested a metal-to-ligand charge transfer (MLCT).

Upon excitation at 430 nm, cluster **1** and the AgNPs showed orange emissions at room temperature at 482 nm (cluster **1**) (Figure 3c) and 560 nm with a shoulder at 579 nm (AgNPs) (Figure 3b). The precise mechanism for the silver nanoparticle fluorescence is unclear, but the polycrystalline structures of silver nanoparticles with numerous ultra-small domains and the particle-supported ultra-small silver nanoclusters could be the possible reasons for the observed luminescence. Moreover, the shoulder observed at 579 nm could be attributed to the presence of silver nanoclusters aggregates with different numbers of atoms.

**Figure 4:**
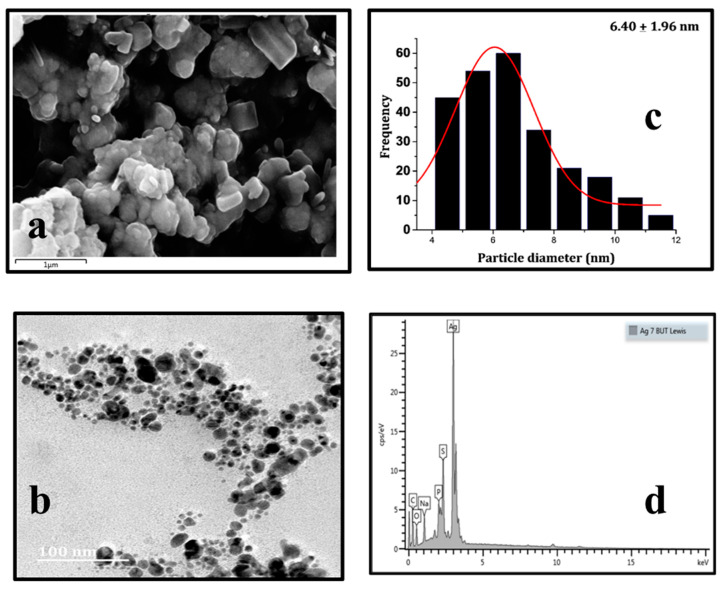
(**a**) SEM micrographs of AgNPs. (**b**) TEM micrographs of AgNPs. (**c**) Particle size distribution of AgNPs. (**d**) EDX spectrum of AgNPs.

The SEM micrograph (Figure 4a) revealed that the nanoparticles formed were polydispersed. Close observation showed that the nanoparticles adopted a spherical morphology with few having irregular shapes. The morphology and particle size investigated using HRTEM (Figure 4b) further confirmed the spherical and non-aggregated nature of the nanoparticles as observed in the SEM micrographs. The histogram of the nanoparticle size distribution obtained from the analysis of the HRTEM micrograph of the synthesized AgNPs (Figure 4c) indicated that the sizes of the nanoparticles range from 4–12 nm with an average of 6 nm.

Energy dispersion X-ray analysis determined the elemental constituents of the synthesized AgNPs. The EDX spectrum (Figure 4d) showed silver as the dominant species with a highly intense peak at a binding energy of 3 keV characteristics of silver. It also showed the presence of phosphorus and sulfur, which indicated the presence of the ligand and suggested that the dithiophosphonate ligand provided a stabilizing effect for the AgNPs. The FTIR analysis further supported this. By comparing the IR spectrum of AgNPs with those of cluster **1** and the ligand (Figure S10, Supplementary Materials), the major vibrational frequencies of the ligand were also observed in the clusters and in the synthesized nanoparticles, which indicated the capacity of the dithiophosphonate ligand to act as a capping agent for the cluster as well as a stabilizing agent to prevent excessive aggregation in the subsequently in situ generated silver nanoparticles.

## 5. Materials and Methods

### 5.1. General Procedures and Instrumentation

All reactions were performed using standard Schleck techniques under a nitrogen atmosphere. All chemicals were purchased from commercial sources and are of analytical grade. THF and DCM were dried using PURE-SOLV solvent purification system, (Innovative Technology, Amesbury, MA, USA, now known as inert, inertcorp.com). [Ag(CH_3_CN)_4_]PF_6_ [53] and NH_4_[S_2_P(4-C_6_H_4_OMe)(OR)] derivatives [54,55] were prepared following literature procedures. NMR spectra were recorded on a Bruker AV-400 instrument (Billerica, MA, USA) operating at 400MHz. The ^1^H and ^31^P-NMR spectra were recorded at 162 MHz. For the ^1^H-NMR, the residual solvent proton was used as a reference (δ, ppm, CDCl_3_, 7.26). For ^31^P-NMR, H_3_PO_4_ (85%) was used as an external reference. The melting point was determined on an Electrothermal 9100 melting point apparatus (Stone, UK) and was uncorrected. The electronic absorption spectra were recorded on a Shimadzu UV-3600 UV-VIS-NIR spectrophotometer (Kyoto, Japan) using quartz cuvettes with a path length of 1 cm in the 200–400 nm for UV and 400–900 nm visible regions. The emission spectra were recorded on Perkin Elmer LS 55 fluorescence spectrometer (Waltham, MA, USA). Electrospray ionization mass spectrum (ESI-MS) for cluster **1** was recorded on Waters Micromass LCT Premier TOF-MS (Milford, MA, USA).

### 5.2. Synthesis of Silver Clusters

#### 5.2.1. Synthesis of cluster **1** ([Ag_7_(H){(S_2_P(4-C_6_H_4_OMe)(OC_4_H_9_)}_6_])

In an oven-dried Schlenk flask [NH_4_]{S_2_P(4-C_6_H_4_OMe)(OC_4_H_9_)} (251 mg, 0.857 mmol) and NaBH_4_ (5.4 mg, 0.142 mmol) were suspended in 15 mL of anhydrous THF. Subsequently, [Ag(CH_3_CN)_4_PF_6_] (417 mg, 1.000 mmol) was added to the reaction mixture and stirred at 0 °C for 3 h. The resulting yellow solution was pumped down under reduced pressure. The residue was dissolved in DCM and washed with deionized water (3 × 15 mL). The DCM fraction was filtered through Celite and dried under reduced pressure. The resulting yellow residue was added to hexane and kept in a refrigerator for several hours. The air and moisture stable yellow powder was obtained by filtration. Yield: 270 mg (92%). Melting point: 112 °C. Anal. Calculated for C_66_H_97_Ag_7_O_12_P_6_S_12_: C, 32.92; H, 4.06; S, 15.98% Found: C, 32.39, H, 4.25. S, 16.22%. ^1^H-NMR (400 MHz, Chloroform-*d*) δ 8.00 (dd, *J* = 13.7, 8.5 Hz, 12H, Ar–H), 6.93 (dd, *J* = 8.8, 3.1 Hz, 12H, Ar–H), 4.11 (q, *J* = 6.8 Hz, 1H, Ag–H), 4.02 (t, *J* = 7.1 Hz, 12H, OCH_2_), 3.85 (s, 18H, OCH_3_), 1.67 (dd, *J* = 8.5, 6.3 Hz, 12H, CH_2_), 1.40 (h, *J* = 7.2 Hz, 12H, CH_2_), 0.91 (t, *J* = 7.3 Hz, 18H, CH_3_). ^31^P-NMR (162 MHz, CDCl_3_) 108.28 (6P, s). ESI-MS (*m*/*z*) [M + Ag]^+^ 2516.00 (calcd 2516.40). UV-VIS [λ_max_ (ε)] 367 nm, 19, 600 M^−1^ cm^−1^. Single-crystal suitable for X-ray analysis was obtained as a colourless rod from a mixture of acetone and hexane.

#### 5.2.2. Synthesis of Cluster **1**_D_ ([Ag_7_(D){(S_2_P(4-C_6_H_4_OMe)(OC_4_H_9_)}_6_])

Cluster **1_D_** was synthesized in 96% yield (280 mg, based on Ag) by following the aforementioned method using NaBD_4_ as a deuteride source instead of NaBH_4_ as a hydride source. Mp: 111 °C, Anal. Calcd for C_66_H_96_DAg_7_O_12_P_6_S_12_: C, 32.92, H, 4.10 S, 15.97 Found: C, 32.40, H, 4.29 S, 16.22. ^1^H-NMR (400 MHz, Chloroform-*d*) δ 8.01 (dd, *J* = 13.7, 8.5 Hz, 12H, Ar–H), 6.95 (dd, *J* = 8.8, 3.1 Hz, 12H, Ar-H), 4.05 (q, *J* = 7.1 Hz, 12H, OCH_2_), 3.85 (s, 18H, OCH_3_), 1.68 (dd, *J* = 8.5, 6.3 Hz, 12H, CH_2_), 1.43 (h, *J* = 7.2 Hz, 12H, CH_2_), 0.92 (t, *J* = 7.3 Hz, 18H, CH_3_). HR-ESI-MS (*m*/*z*) [M + Ag]^+^ 2517.00 (calcd 2517.45).

#### 5.2.3. Synthesis of Cluster **2** ([Ag_7_(H){S_2_P(4-C_6_H_4_OMe)(OC_3_H_7_)}_6_])

Cluster **2** was prepared in a similar manner to cluster **1**. Yield: 125 mg (90%, based on Ag) Mp: 108 °C, Anal. Calcd for C_60_H_85_Ag_7_O_12_P_6_S_12_: C, 31.01; H, 3.69; S, 16.55. Found: C, 30.80; H, 3.83; S, 16.97. ^1^H-NMR (400 MHz, Chloroform-*d*) δ 7.91 (dd, *J* = 13.8, 8.4 Hz, 12H, Ar–H), 6.85 (dd, *J* = 7.8 Hz, 12H, Ar–H), 3.90 (t, *J* = 9.7 Hz, 12H, OCH_2_), 3.77 (s, 18H, OCH_3_), 3.77 (q, *J* = 2.6, 2.0 Hz, 1H, Ag–H), 1.66–1.61 (m, 12H, CH_2_), 0.86 (t, *J* = 7.4 Hz, 18H, CH_3_). ^31^P-NMR (162 MHz, CDCl_3_) 108.28 (6P, s). ESI-MS (*m*/*z*) [M + Ag]^+^ 2432.35 (calcd 2432.33). 

#### 5.2.4. Synthesis of Cluster **2**_D_ ([Ag_7_(D) {S_2_P(4-C_6_H_4_OMe)(OC_3_H_7_)}_6_])

Cluster **2_D_** was obtained in 90% yield (0.20 g, based on Ag) by following the aforementioned method using NaBD_4_ as a deuteride source instead of NaBH_4_ as a hydride source. Mp: 110 °C, Anal. Calcd for C_60_H_84_DAg_7_O_12_P_6_S_12_. C, 31.00; H, 3.73; S, 16.55 Found: C_60_H_84_DAg_7_O_12_P_6_S_12_. C, 30.42; H, 3.80; S, 16.85 ^1^H-NMR (400 MHz, Chloroform-d) δ 7.89 (dd, *J* = 13.8, 8.4 Hz, 12H, Ar–H), 6.84 (dd, *J* = 7.8 Hz, 12H, Ar–H), 3.90 (d, *J* = 9.7 Hz, 12H, OCH_2_), 3.77 (s, 18H, OCH_3_), 1.65 (m, 12H, CH_2_),0.85 (t, *J* = 7.4 Hz, 18H, CH_3_). ESI-MS (*m*/*z*) [M + Ag]^+^ 2433.31 (calcd 2433,34).

#### 5.2.5. Synthesis of Cluster **3** ([Ag_7_(H){S_2_P(4-C_6_H_4_OMe)(OCH_2_C_6_H_5_)}_6_])

Cluster **3** was prepared in a similar manner to **1**. Yield: 101 mg (56%) ^1^H-NMR (400 MHz, Chloroform-*d*): δ (ppm), 8.02 (dd, *J* = 9.01, 12H, Ar–H), 7.39 (d, *J* = 8.10 Hz, 12H, Ar–H), 7.35 (d, *J* = 7.56 Hz, 12H, Ar–H), 6.95 (dd, *J* = 8.78, 12H, Ar–H), 5.29 (s, 12H, CH_2_), 4.71 (bs, 1H, Ag–H), 3.85 (s, 18H, OCH_3_), ^31^P-NMR (162 MHz, CDCl_3_) 101.68 (6P, s). ESI-MS (*m*/*z*) [M + Ag]^2+^ 1467.92 (Calcd 1468.00). Single crystals for X-ray analysis were obtained as yellow blocks by vapour diffusion of hexane into a concentrated DCM solution.

#### 5.2.6. Synthesis of Cluster **4** ([Ag_7_(H){S_2_P(4-C_6_H_4_OMe)(OC_5_H_11_)}_6_])

Cluster **4** was prepared by adopting a procedure similar to that of cluster **1**. Yield: 123 mg (76%). Melting point: 121 °C. ^1^H-NMR (400 MHz, DMSO-*d*_6_) δ 7.76 (dd, *J* = 13.6, 8.5 Hz, 12H, Ar–H), 7.09 (dd, *J* = 8.7, 3.2 Hz, 12H, Ar–H), 4.01 (bs, 1H, Ag–H) 4.46 (dp, *J* = 11.6, 5.8 Hz, 6H, OCH), 3.83 (s, 18H, OCH_3_), 1.66 (dq, *J* = 9.2, 7.1 Hz, 24H, CH_2_), 0.93 (t, *J* = 7.4 Hz, 36H, CH_3_), HR-ESI-MS (*m*/*z*) [M + Ag]^+^ 2600.5259 (calcd 2600.20). A single crystal for X-ray analysis was obtained as a colourless rod by slow evaporation from a mixture of benzene and hexane.

### 5.3. Characterization of AgNP

The morphology of the AgNPs was examined using electron microscopy. The high-resolution transmission electron microscopy (HRTEM) analysis was conducted on a JEOL 2100 (Tokyo, Japan) high-resolution transmission electron microscope. The scanning electron microscopy analysis was conducted on a Zeiss Ultra Plus field emission gun scanning electron microscope (FEG-SEM) equipped with an energy dispersive X-ray (EDX) detector (Jena, Germany). The HRTEM images of the AgNPs were obtained by using 5 µL of each sample deposited on a copper TEM grid. The particle size was obtained by measuring the diameters of 150 AgNPs in the HTEM micrographs using ImageJ 1.42 software (NIH, Bethesda, MD, USA), and the obtained data were processed on OriginPro 8 software (OriginLab Corporation, Northampton, MA, USA). Similarly, the SEM images were obtained by depositing a dried product of AgNPs samples on a conductive carbon tape stuck to aluminum stubs. To minimize charging, the sample was coated with gold with the aid of a sputter coater.

### 5.4. Catalytic Reduction of 4-NP by Cluster ***1*** and AgNPs

For the reduction of 4*-*NP, the procedure employed a standard catalytic test, as reported by Kästner et al. [46]. An aqueous solution of 4-NP (2 mL, 10^−4^ M) was mixed with a freshly prepared aqueous solution of NaBH_4_ (1 mL, 10^−4^ M) in a quartz cell (1.0 cm path length and 4.5 mL volume). Cluster **1** (2.5 µL, 6 nmol) was dispersion in water and was added to the solution. The reduction was monitored at room temperature using a UV-vis spectrophotometer scanning over a range of 250–550 nm with a successive 1 min interval at room temperature. The apparent rate constant (Kapp) of the catalytic reaction was determined by measuring the absorbance at 400 nm. This procedure was repeated using AgNPs as a catalyst.

### 5.5. X-ray Crystallographic Determination

Experimental. The single crystals of **1**, **3**, **4** were selected and mounted on a MITIGEN holder in paratone oil on a Bruker SMART APEX2 area detector diffractometer (Billerica, MA, USA). During data collection, the crystal was kept at T = 100(2) K. Using Olex2, [56] the structure was solved with the SHELXS-2013 [57] structure solution program, using the direct solution method. The model was refined with SHELXL [58] using Least Squares minimization. All non-hydrogen atoms were refined anisotropically. Most hydrogen atom positions were calculated geometrically and refined using the riding model, but some hydrogen atoms were freely refined.

## 6. Conclusions

The study demonstrated the synthesis of new air and moisture stable Ag_7_ clusters ligated by dithiophosphonate ligands and characterized by single-crystal XRD, NMR, UV-Vis and luminescence spectroscopy. The crystal structure revealed that the dithiophosphonate (DTP) ligands were coordinated in a tetra-connective tetra-metallic (µ_2_, µ_2_) mode representing the first of this binding mode for DTP ligands and the first high nuclearity silver cluster stabilized by this ligand type. Cluster **1**, in the presence of excess borohydride, yielded silver nanoparticles and simultaneous reduction of 4-NP to 4-AP. The obtained nanoparticles were characterized by TEM, SEM, UV-Vis and luminescence spectroscopy which revealed that the AgNPs are monolithic and luminescent, with an average size diameter of 6.4 nm. To establish the mechanism behind the catalytic activity of cluster **1**, AgNPs were first synthesized from cluster **1** and subsequently applied as a catalyst for the reduction of 4-NP. The cluster showed an excellent ability to reduce 4-NP, and expectedly the AgNPs also showed an excellent ability under the same conditions. We concluded the size and dispersion of the AgNPs are key components to account for their activity. The mechanism of reduction of 4-NP using silver hydride cluster, however, is not fully understood, but it is believed that the presence of reactive Ag-H species makes the transfer of hydride ion to the intermediate (4-(hydroxyamino)phenol) possible.

## Data Availability

CCDC numbers 1980078 contain the supplementary crystallographic data for this paper. These data can be obtained free of charge via www.ccdc.cam.ac.uk/data_request/CIF, (access on 15 July 2022) by emailing data_request@ccdc.cam.ac.uk, or by contacting The Cambridge Crystallographic Data Centre, 12 Union Road, Cambridge CB2 1EZ, UK; fax: +44-1223-336033. Crystallographic details.

## Supplementary Materials

The following supporting information can be downloaded at: https://www.mdpi.com/article/10.3390/molecules27165223/s1, Figure S1: ^1^H-NMR spectrum for cluster **1**; Figure S2: ^31^P-NMR spectrum for cluster **1**; Figure S3: ^31^P-NMR spectrum for cluster **2**; Figure S4: HRMS for cluster **1**; Figure S5: HRMS for cluster **1_D_**; Figure S6: HRMS for cluster **2**; Figure S7: HRMS for cluster **2_D_**; Figure S8: HRMS for cluster **4**; Figure S9: Mass spec of cluster **3**; Figure S10: Comparative FT-IR spectra of DTP, cluster **1** and AgNPs; Figure S11: Mapping of the constituent elements of AgNps; Figure S12: Percentage catalytic reduction achieved with the AgNPs over 5 cycles; Table S1: Selected crystallographic data for 1; Table S2: Fractional Atomic Coordinates (×104) and Equivalent Isotropic Displacement Parameters (Å2 × 103) for Cluster **1**; Table S3: Anisotropic Displacement Parameters (Å2 × 103) for Cluster **1**; Table S4: Bond Lengths for Cluster **1**; Table S5: Bond Angles for Cluster **1**; Table S6: Torsion Angles for Cluster **1**; Table S7: Hydrogen Atom Coordinates (Å × 104) and Isotropic Displacement Parameters (Å2 × 103) for Cluster **1**; Table S8: EDX elemental constituent of AgNPs.

## Sample Availability

Samples are available from authors pending decomposition over time.
