# Supplementary material for: Heptanuclear Silver Hydride Clusters as Catalytic Precursors for the Reduction of 4-Nitrophenol
Source: Molecules. 2022 Aug 16;27(16):5223. doi: 10.3390/molecules27165223 (PMC9415167; doi:10.3390/molecules27165223)
Supplement: Supplementary file 1 [file molecules-27-05223-s001.zip › molecules-1816686-supplementary.pdf]

molecules

MDPI

Supplementary Materials

Heptanuclear Silver Hydride Clusters as Catalytic Precursors for the Reduction of 4-Nitrophenol

Tunde L. Yusuf [1], Segun A. Ogundare [1,2], Michael N. Pillay [1] and Werner E. van Zyl [1,*]

[1] School of Chemistry and Physics, University of KwaZulu-Natal, Westville Campus, Chiltern Hills, Durban 4000, South Africa
[2] Department of Chemical Sciences, Olabisi Onabanjo University, Ago-Iwoye 2002, Nigeria
* Correspondence: vanzylw@ukzn.ac.za

Content	Page
^{1}H NMR spectra	2
^{31}P NMR spectra	3
High resolution mass spectra	4-6
FTIR spectra	7
EDX mapping of AgNPs	8
Catalytic reduction cycles	9
Xray parameters	9
Fractional coordinates	10
Anisotropic parameters	12
Bond lengths	14
Bond angles	15
Torsion angles	18
Hydrogen bonding	20
EDX analysis for AgNPs	22

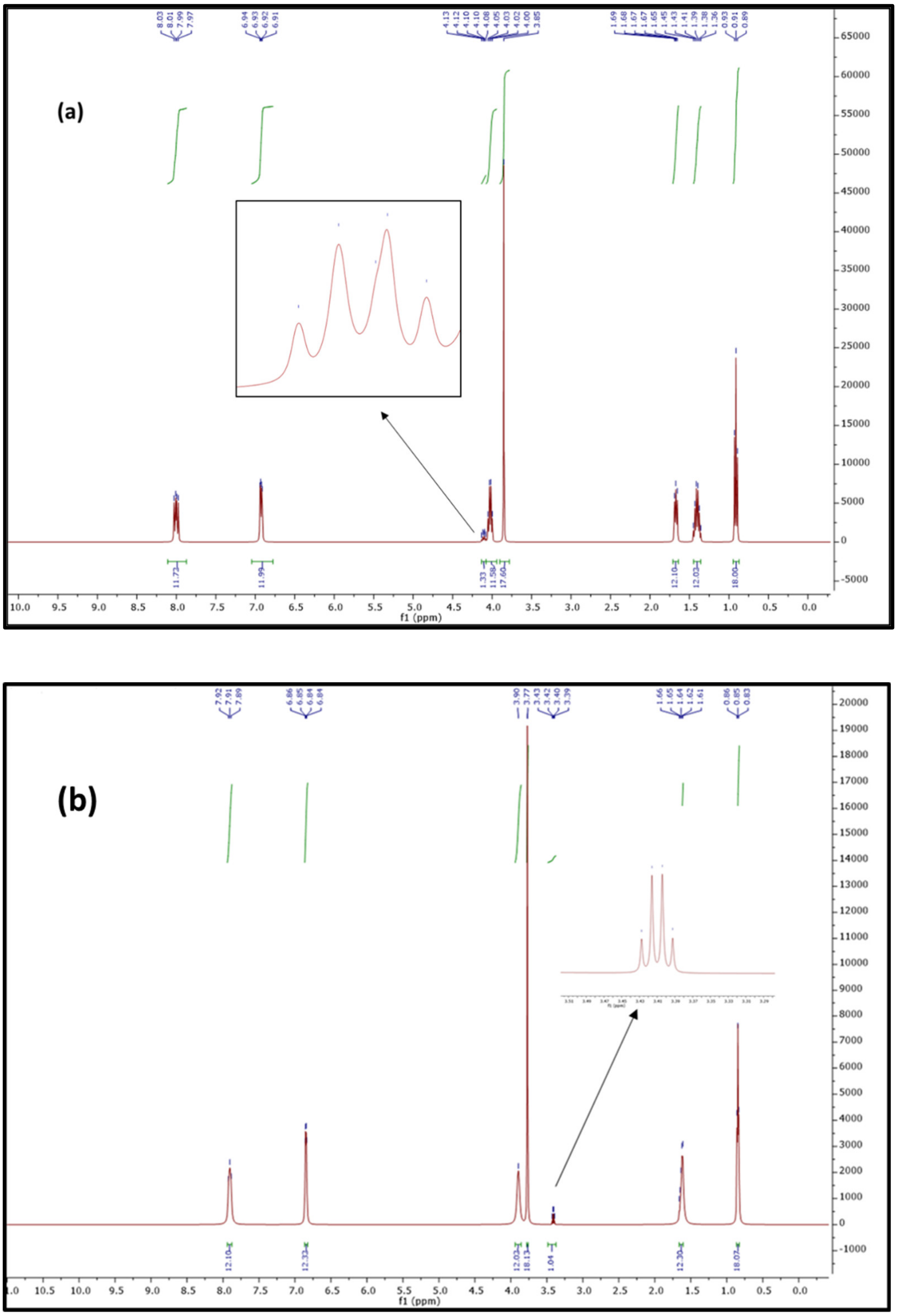

Figure S1. ^{1}H NMR spectra for clusters **1(a)** and **2(b).**

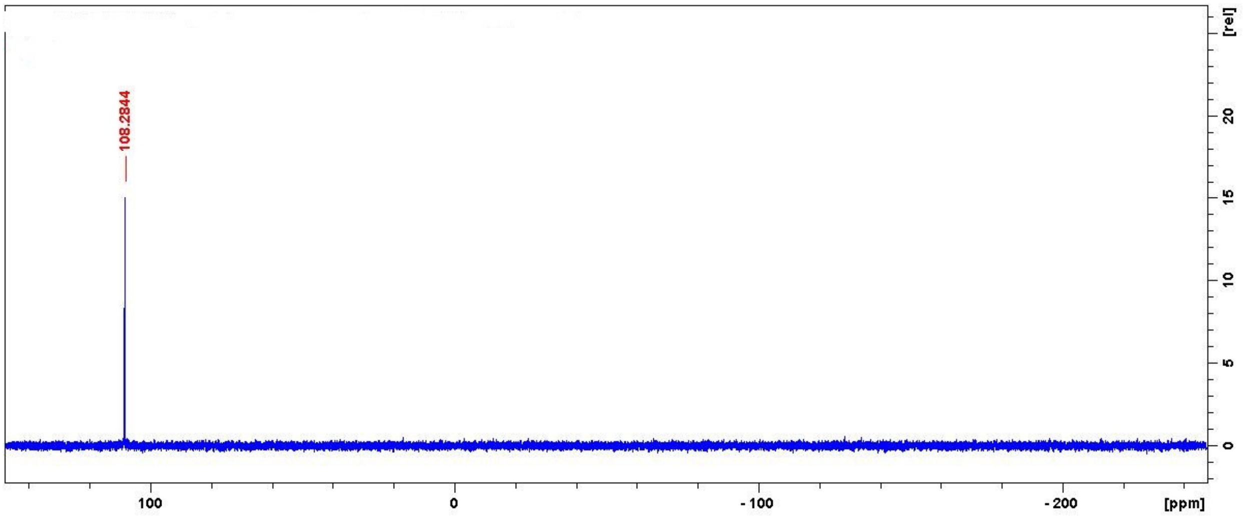

Figure S2. ^{31}P NMR spectrum for cluster **1**

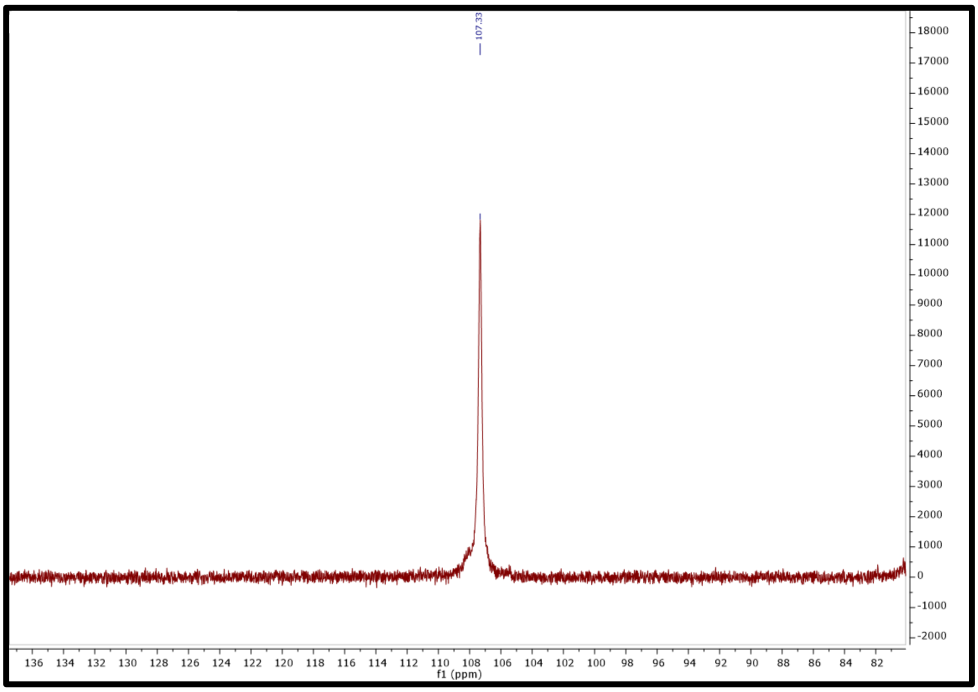

Figure S3. ^{31}P NMR spectrum for cluster **2**

Elemental Composition Report **Page 1**

Single Mass Analysis
Tolerance = 5.0 PPM / DBE: min = -1.5, max = 500.0
Element prediction: Off
Number of isotope peaks used for i-FIT = 2

Monoisotopic Mass, Odd and Even Electron Ions
368 formula(e) evaluated with 1 results within limits (up to 20 closest results for each mass)
Elements Used:
C: 66-70 H: 95-100 O: 12-12 P: 5-10 S: 12-12 107Ag: 0-8 109Ag: 0-8

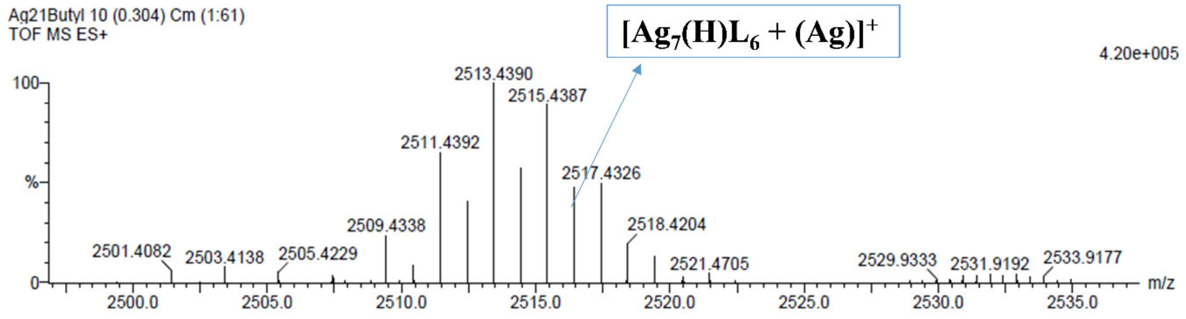

Minimum:				-1.5			
Maximum:		5.0	5.0	500.0			
Mass	Calc. Mass	mDa	PPM	DBE	i-FIT	i-FIT (Norm)	Formula
2509.4338	2509.4377	-3.9	-1.6	18.0	62.3	0.0	C66 H96 O12 P6 S12 107Ag6 109Ag2

Figure S4: HRMS for cluster **1**

Elemental Composition Report **Page 1**

Single Mass Analysis
Tolerance = 5.0 PPM / DBE: min = -1.5, max = 500.0
Element prediction: Off
Number of isotope peaks used for i-FIT = 2

Monoisotopic Mass, Odd and Even Electron Ions
20 formula(e) evaluated with 1 results within limits (up to 20 closest results for each mass)
Elements Used:
C: 65-70 H: 95-110 O: 12-12 P: 6-6 S: 12-12 107Ag: 0-6 109Ag: 0-2

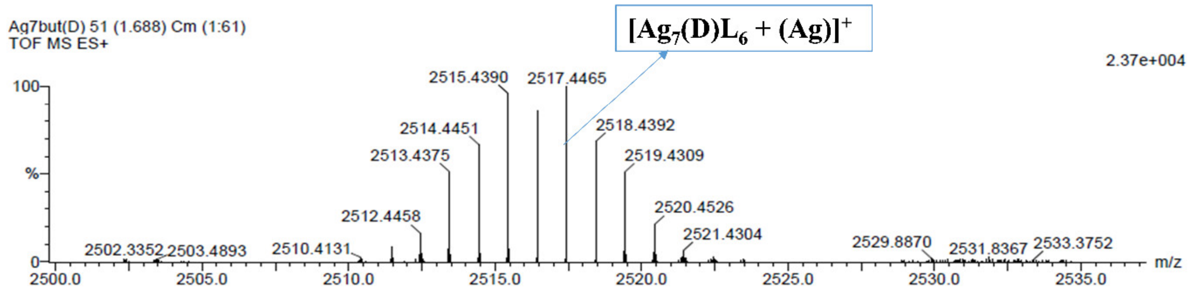

Minimum:				-1.5			
Maximum:		5.0	5.0	500.0			
Mass	Calc. Mass	mDa	PPM	DBE	i-FIT	i-FIT (Norm)	Formula
2511.4565	2511.4533	3.2	1.3	17.0	116.4	0.0	C66 H98 O12 P6 S12 107Ag6 109Ag2

Figure S5. HRMS for cluster **1**$_D$

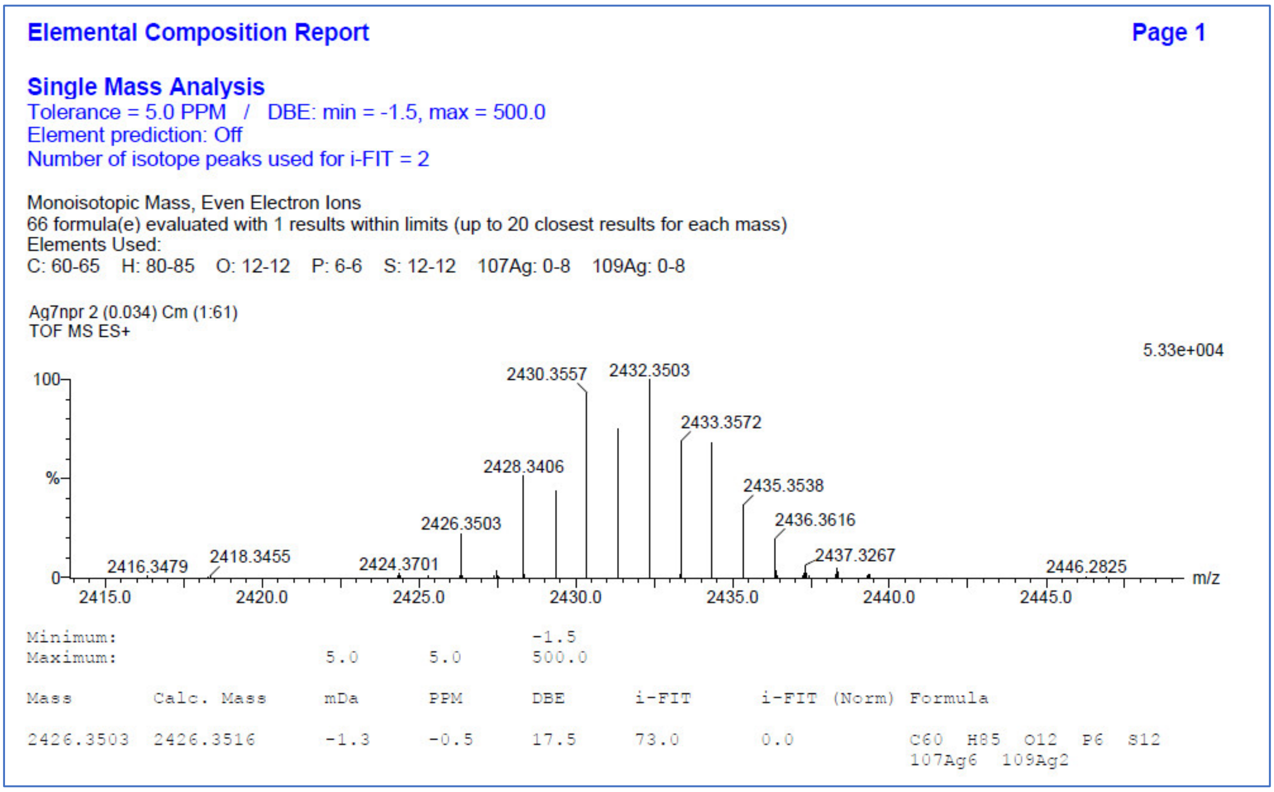

Elemental Composition Report Page 1

Single Mass Analysis
Tolerance = 5.0 PPM / DBE: min = -1.5, max = 500.0
Element prediction: Off
Number of isotope peaks used for i-FIT = 2

Monoisotopic Mass, Even Electron Ions
66 formula(e) evaluated with 1 results within limits (up to 20 closest results for each mass)
Elements Used:
C: 60-65 H: 80-85 O: 12-12 P: 6-6 S: 12-12 107Ag: 0-8 109Ag: 0-8

Ag7npr 2 (0.034) Cm (1:61)
TOF MS ES+

Minimum:				-1.5			
Maximum:		5.0	5.0	500.0			
Mass	Calc. Mass	mDa	PPM	DBE	i-FIT	i-FIT (Norm)	Formula
2426.3503	2426.3516	-1.3	-0.5	17.5	73.0	0.0	C60 H85 O12 P6 S12 107Ag6 109Ag2

Figure S6. HRMS for cluster **2**

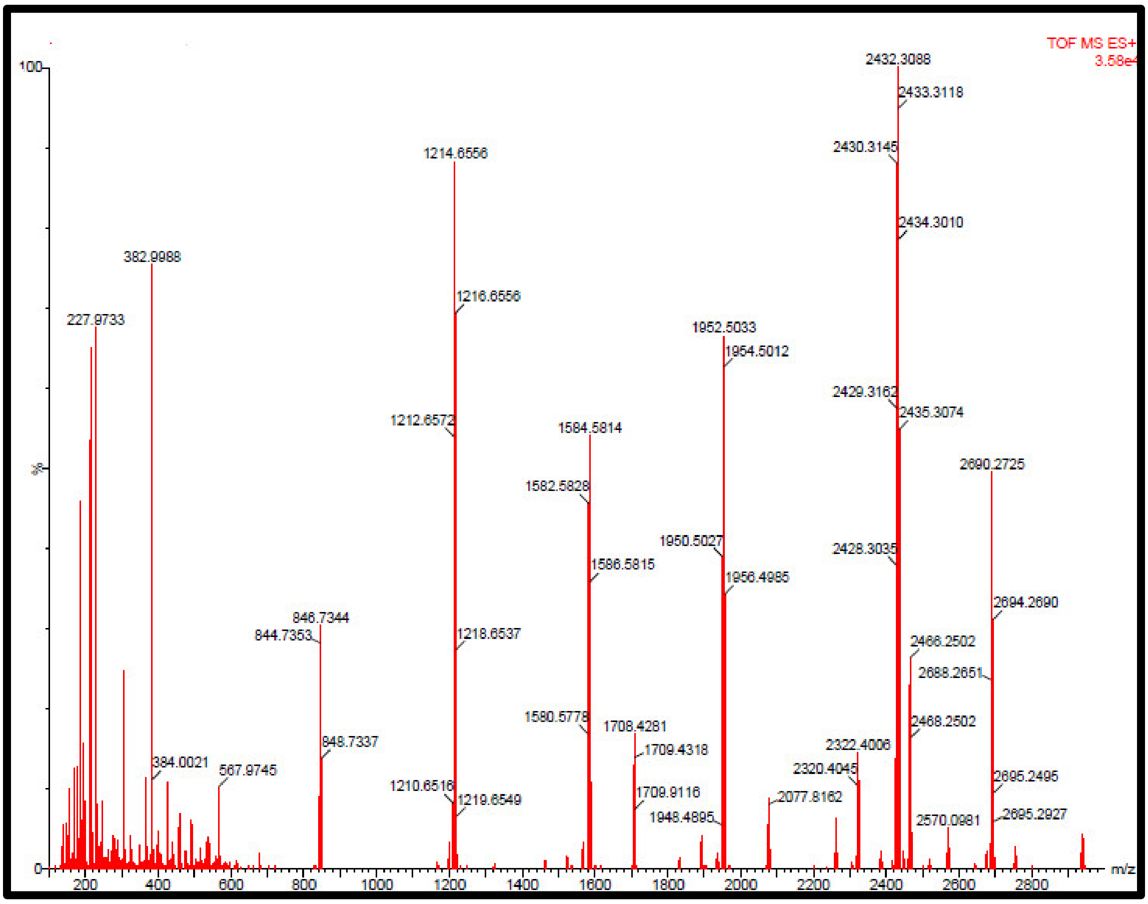

Figure S7. HRMS for cluster **2**D

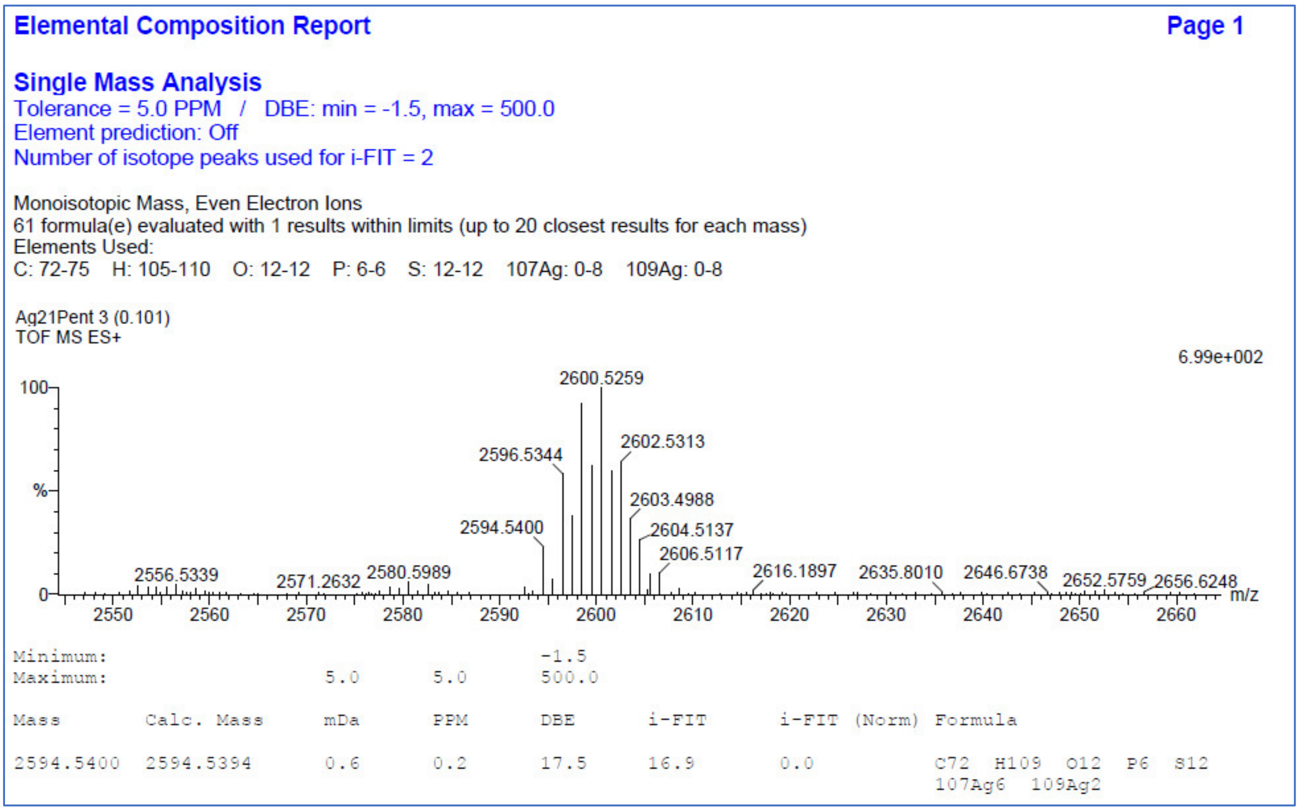

Elemental Composition Report **Page 1**

Single Mass Analysis
Tolerance = 5.0 PPM / DBE: min = -1.5, max = 500.0
Element prediction: Off
Number of isotope peaks used for i-FIT = 2

Monoisotopic Mass, Even Electron Ions
61 formula(e) evaluated with 1 results within limits (up to 20 closest results for each mass)
Elements Used:
C: 72-75 H: 105-110 O: 12-12 P: 6-6 S: 12-12 107Ag: 0-8 109Ag: 0-8

Minimum:				-1.5			
Maximum:		5.0	5.0	500.0			
Mass	Calc. Mass	mDa	PPM	DBE	i-FIT	i-FIT (Norm)	Formula
2594.5400	2594.5394	0.6	0.2	17.5	16.9	0.0	C72 H109 O12 P6 S12 107Ag6 109Ag2

Figure S8. HRMS for **cluster 4**

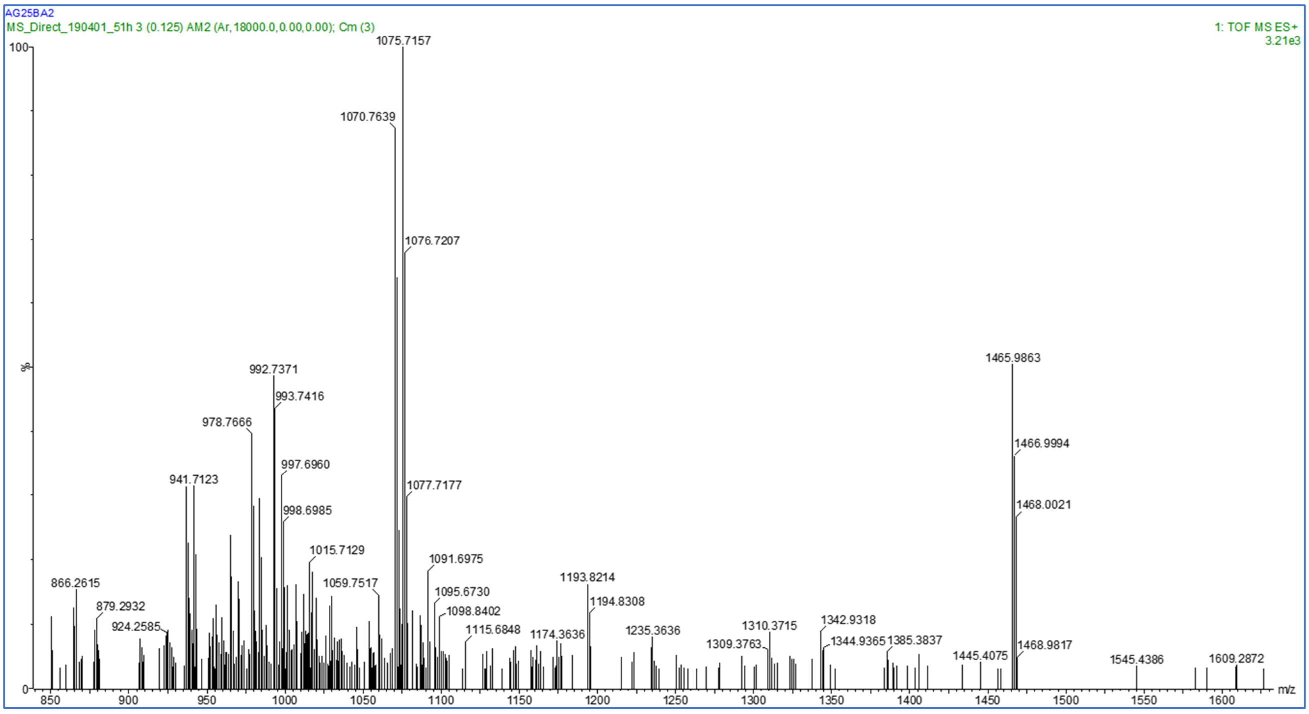

Figure S9: Mass spectrum for **cluster 3**

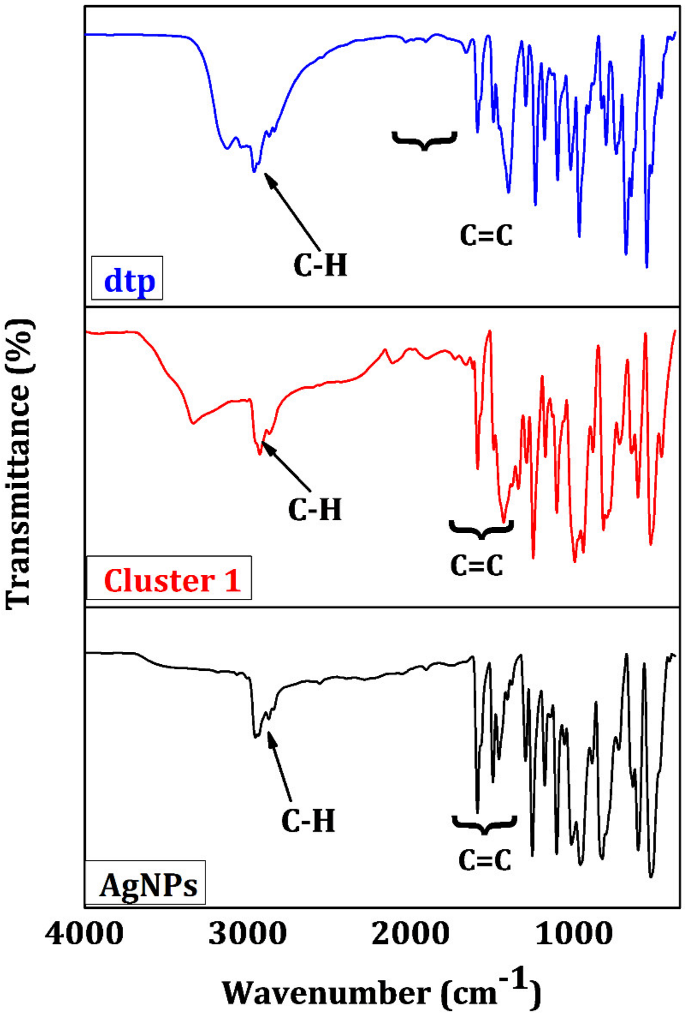

Figure S10. Comparative FT-IR spectra of DTP, Cluster **1** and AgNPs

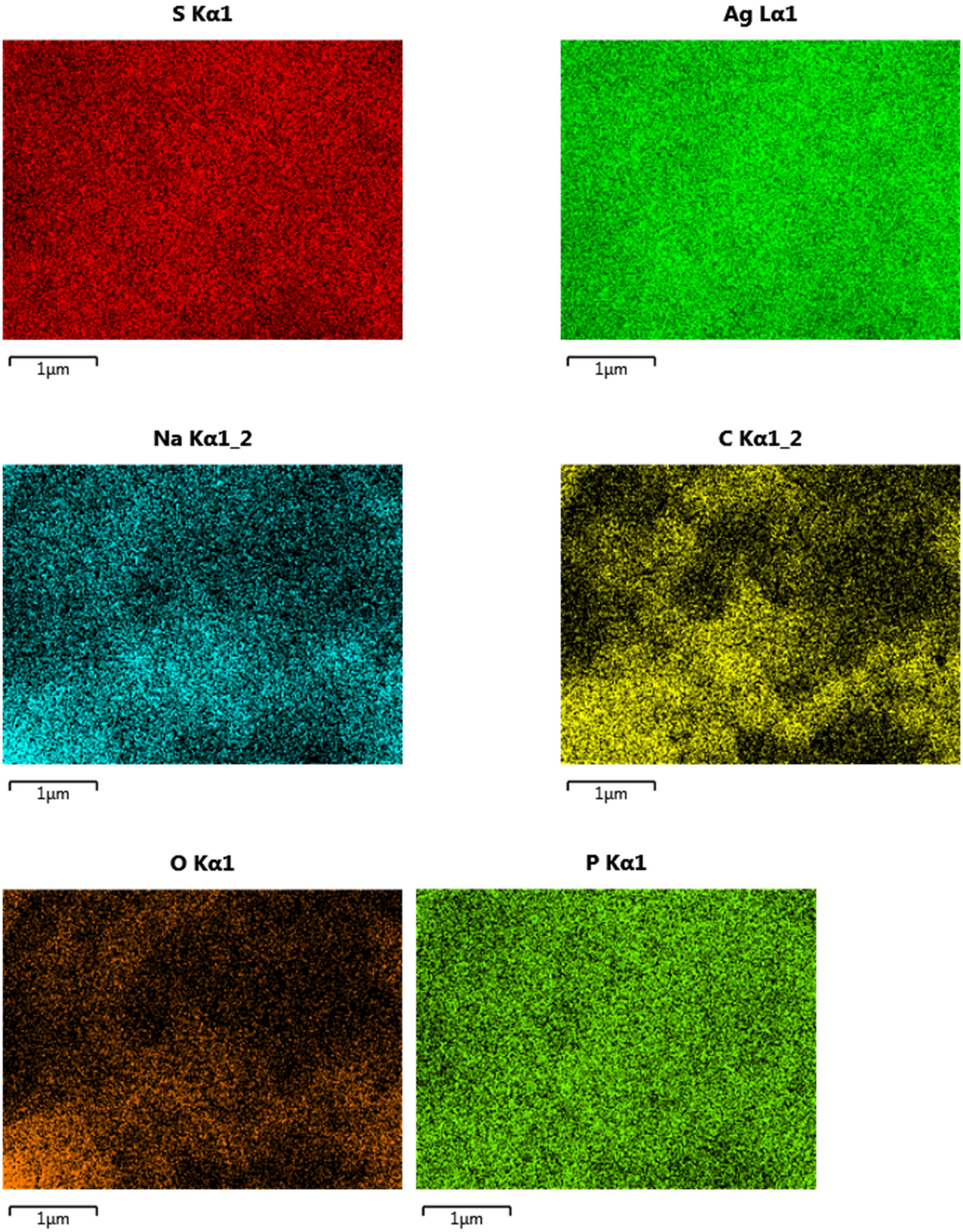
S Kα1
1μm
Ag Lα1
1μm
Na Kα1_2
1μm
C Kα1_2
1μm
O Kα1
1μm
P Kα1
1μm

Figure S11. Mapping of the constituent elements of AgNPs

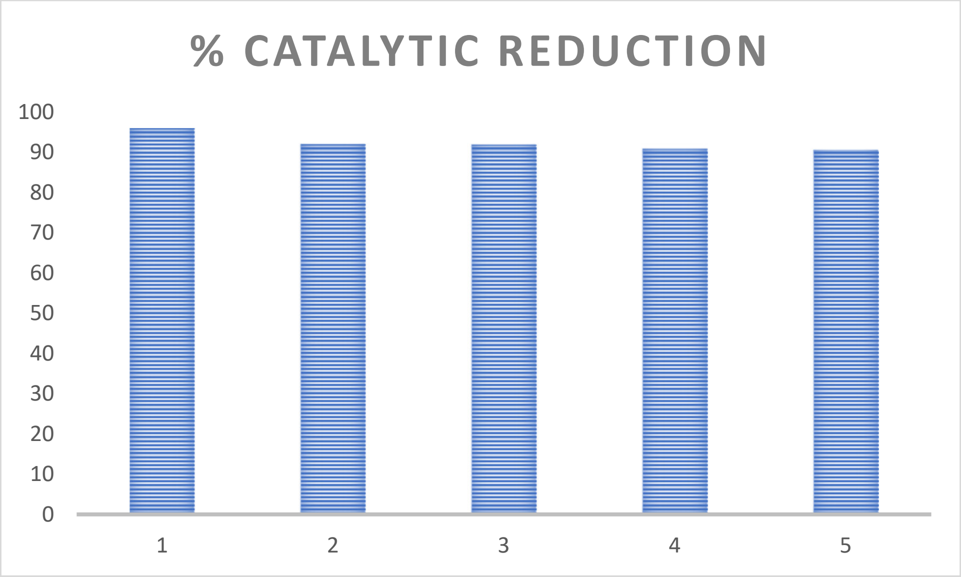

Figure S12. Percentage catalytic reduction achieved with the AgNPs over 5 cycles.

Table S1. Selected crystallographic data for **1**

	**1**
Formula	$C_{66}H_{97}Ag_7O_{12}P_6S_{12}$
Formula weight	2408.2
Crystal System	Triclinic
Space Group	*P*(-)1
a/Å	12.4722(10)
b/Å	13.5645(11)
c/Å	15.2623(12)
α, deg	111.838(2)
β, deg	103.657(2)
γ, deg	103.016(2)
V/Å^3	2184.2(3)
Z	1
F(000	1896
$\rho_{calcd,}$/ g cm^{-3}	1.831
μ/mm^{-1}	1.986
T, K	100(2)
Wavelength/Å	0.71073
Measured Refl.	27974
Independent Refl.	7971 [R_{int} = 0.0223, R_{sigma} = 0.0215]
Restraints/Parameters	457/695
R1, wR2 [I>2σ(I)]	0.0647, 0.1615
R1, wR2 (all data)	0.0866, 0.1819
GooF	1.025
Largest Peak, Deepest Hole	1.869, -1.022

Table S2. Fractional Atomic Coordinates (×10[4]) and Equivalent Isotropic Displacement Parameters (Å[2]×10[3]) for Cluster 1. U_{eq} is defined as 1/3 of the trace of the orthogonalised U_{IJ} tensor.

Atom	*x*	*y*	*z*	U(eq)
S4	2896.9(17)	2329.9(15)	2036.1(13)	47.7(4)
S2	-460(2)	1946.8(17)	2339.6(17)	59.0(5)
P3	3652.1(16)	2428.3(14)	1026.4(14)	42.5(4)
P2	-1367.9(19)	2699.5(15)	-246.0(16)	47.2(5)
P1	251.0(18)	1301.2(16)	3220.1(15)	50.4(5)
S6	2744.4(19)	1353.2(17)	-435.2(15)	54.2(5)
S3	-2452.5(19)	1214.5(16)	-485.5(19)	58.2(5)
S5	367.8(19)	2941.9(16)	127.7(19)	58.9(6)
S1	751(2)	-31.4(19)	2590.9(16)	62.0(6)
O7	4897(5)	2277(4)	1342(4)	51.6(13)
O8	-1814(7)	2982(6)	-1201(6)	81(2)
O6	5390(8)	7158(7)	1636(7)	35.6(18)
O1	1329(7)	2279(7)	4153(7)	111(3)
C22	4960(8)	1211(7)	1315(7)	59.3(19)
O4	-1623(16)	6635(10)	3076(12)	186(7)
C24	6551(12)	2160(12)	2923(10)	103(4)
C23	6140(9)	1368(8)	1878(9)	76(2)
C50	-1540(20)	7564(19)	2870(20)	195(11)
Ag6	921.5(10)	2430.1(9)	1457.9(8)	42.6(3)
Ag7	714.3(13)	1385.1(11)	-940.1(11)	50.6(3)
Ag2	-1999.0(12)	-374.5(11)	-1728.2(11)	48.4(3)
Ag4	-1842(4)	609(3)	760(4)	51.2(10)
Ag1	-1380.8(11)	-221.8(8)	-1005.5(9)	47.5(3)
Ag3	-1219.9(11)	355.0(9)	422.9(9)	47.7(3)
Ag1A	532.1(11)	835.2(9)	-701.2(8)	47.0(3)
Ag5	541.4(12)	1593.6(10)	1046.1(9)	52.7(3)
C27	3520(20)	4170(15)	505(16)	48(5)
C28	3890(16)	5274(13)	637(13)	48(5)
C29	4913(11)	6031(10)	1419(9)	30(2)
C30	5580(12)	5719(10)	2066(11)	46(3)
C31	5200(12)	4616(9)	1921(11)	44(3)
C26	4164(16)	3846(13)	1143(12)	29(3)
C8	-716(14)	1847(13)	4687(10)	62(4)
C7	-790(17)	1042(13)	3772(12)	44(5)
C12	-1629(17)	-22(14)	3360(13)	62(6)
C11	-2390(20)	-290(16)	3835(15)	79(7)

C10	-2266(16)	512(13)	4781(12)	60(4)
C9	-1448(16)	1600(14)	5192(13)	73(5)
C3	-1263(14)	506(16)	5109(12)	76(5)
C4	-2433(13)	-52(12)	4479(11)	56(4)
C5	-2774(15)	-206(14)	3491(12)	55(4)
C6	-1919(12)	213(13)	3162(12)	44(4)
C1	-754(15)	781(15)	3784(10)	36(3)
C2	-424(14)	916(17)	4758(10)	67(4)
C33	4800(20)	4713(16)	2323(16)	88(6)
C32	4090(20)	3910(20)	1353(17)	60(7)
C37	3790(30)	4280(20)	630(20)	72(7)
C36	4150(20)	5428(19)	872(17)	67(6)
C35	4890(20)	6211(18)	1835(15)	78(5)
C34	5230(20)	5856(16)	2568(17)	102(6)
C40	-2119(11)	2233(10)	-2216(8)	29(2)
C42	-1921(11)	4026(11)	-2481(10)	36(3)
C41	-2624(13)	2788(14)	-2822(12)	56(4)
C43	-2480(30)	4481(15)	-3173(15)	103(10)
C25	5830(15)	1908(16)	3517(11)	131(5)
C39	4685(12)	7505(11)	958(11)	35(3)
O5	5312(14)	7362(13)	2182(11)	90(4)
C38	4972(19)	7764(16)	1415(13)	70(5)
C18	2086(18)	3291(16)	4371(14)	72(5)
C19	2250(30)	4250(20)	5310(20)	114(8)
C20	1180(30)	4550(30)	5330(30)	150(10)
C21	530(40)	4900(40)	4650(30)	184(17)
C17	4600(30)	5090(40)	5310(30)	183(16)
C16	3540(30)	4280(20)	5130(30)	164(12)
C15	3340(30)	3150(20)	4900(20)	137(9)
C14	2220(20)	2369(19)	4630(20)	92(6)
C13	-4328(19)	-990(20)	4300(20)	79(6)
O3	-3189(16)	-384(17)	4927(13)	78(4)
O2	-2954(15)	356(16)	5326(14)	76(4)
C45	-1684(13)	3825(8)	1534(8)	105(4)
C46	-1741(13)	4757(9)	2285(11)	137(5)
C47	-1539(14)	5764(10)	2277(11)	120(4)
C48	-1258(10)	5843(8)	1515(8)	84(3)
C49	-1234(10)	4915(7)	744(9)	84(3)
C44	-1536(8)	3806(6)	657(7)	59(2)

C0AA	-3670(30)	-800(20)	5030(30)	101(10)

Table S3. Anisotropic Displacement Parameters (Å^2×10^3) for Cluster 1. The Anisotropic displacement factor exponent takes the form: $-2\pi^2[h^2a^{*2}U_{11}+2hka^*b^*U_{12}+\ldots]$.

Atom	U_{11}	U_{22}	U_{33}	U_{23}	U_{13}	U_{12}
S4	52.1(10)	37.4(9)	42.7(9)	12.1(7)	20.1(8)	3.0(8)
S2	78.6(14)	44.8(10)	63.3(12)	21.9(9)	46.2(11)	20.5(10)
P3	42.9(10)	29.7(8)	52.6(10)	18.6(8)	20.2(8)	4.7(7)
P2	63.3(12)	28.9(8)	66.6(12)	29.8(9)	37.0(10)	17.1(8)
P1	51.4(11)	38.4(10)	46.2(10)	6.3(8)	22.5(9)	5.5(8)
S6	58.1(11)	46.6(10)	51.5(10)	16.2(8)	31.7(9)	3.9(9)
S3	61.9(12)	37.1(9)	97.9(16)	42.0(10)	44.8(12)	19.0(9)
S5	63.1(12)	32.7(9)	97.2(16)	39.1(10)	41.4(12)	14.4(9)
S1	84.3(15)	54.5(12)	49.4(11)	17.8(9)	31.7(11)	29.1(11)
O7	50(3)	41(3)	77(4)	35(3)	29(3)	16(2)
O8	94(5)	61(4)	118(6)	62(4)	50(4)	29(4)
O6	36(2)	35(2)	36(2)	16.2(12)	12.1(11)	12.2(11)
O1	57(4)	78(5)	106(6)	-25(4)	10(4)	-8(4)
C22	78(5)	40(4)	74(5)	29(4)	40(4)	26(4)
O4	350(20)	125(9)	229(14)	110(10)	222(15)	154(12)
C24	95(8)	101(8)	109(6)	39(6)	16(5)	65(7)
C23	69(5)	54(5)	115(6)	46(5)	36(5)	21(4)
C50	270(30)	220(20)	310(30)	220(20)	200(20)	170(20)
Ag6	52.8(6)	26.9(5)	46.7(6)	12.9(4)	27.1(5)	7.7(4)
Ag7	69.0(9)	37.3(7)	60.2(8)	27.6(6)	39.4(7)	17.2(6)
Ag2	51.9(7)	37.7(6)	51.6(8)	20.3(6)	21.5(6)	4.8(5)
Ag4	62(3)	34(2)	67(3)	20.8(19)	42(2)	16.8(19)
Ag1	60.4(7)	26.6(5)	50.8(7)	13.7(5)	22.0(6)	10.5(5)
Ag3	55.0(7)	31.7(5)	55.1(6)	18.3(5)	26.5(5)	7.4(5)
Ag1A	63.3(7)	28.9(5)	46.4(6)	16.1(5)	25.1(5)	7.3(5)
Ag5	73.1(8)	34.0(7)	52.2(7)	19.1(5)	28.7(6)	14.4(6)
C27	43(8)	36(6)	56(8)	25(6)	7(6)	1(5)
C28	45(7)	34(6)	56(8)	25(5)	3(6)	2(5)
C29	33(5)	26(4)	38(5)	17(4)	20(5)	11(4)
C30	39(6)	26(5)	57(7)	20(5)	1(5)	-1(4)
C31	39(6)	25(5)	53(7)	17(5)	-1(6)	2(4)

C26	24(6)	19(5)	40(6)	12(5)	14(5)	1(4)
C8	66(8)	50(7)	51(6)	9(6)	23(6)	9(6)
C7	61(9)	31(8)	44(6)	21(5)	20(6)	14(6)
C12	90(11)	42(8)	46(7)	17(6)	34(7)	1(7)
C11	104(13)	59(8)	66(9)	25(7)	47(9)	-1(8)
C10	61(9)	67(8)	53(8)	29(6)	26(7)	17(7)
C9	78(10)	67(8)	65(8)	19(6)	37(7)	16(7)
C3	83(8)	98(12)	54(7)	42(7)	33(6)	21(7)
C4	82(8)	48(9)	54(7)	30(6)	36(6)	26(7)
C5	66(8)	54(8)	53(7)	28(6)	31(6)	21(6)
C6	58(7)	39(7)	41(6)	20(5)	21(5)	19(6)
C1	54(7)	27(7)	36(6)	16(5)	23(5)	20(6)
C2	65(8)	92(11)	45(6)	36(6)	22(5)	19(7)
C33	99(10)	72(7)	86(8)	47(6)	13(6)	20(6)
C32	56(11)	64(9)	78(9)	49(7)	28(7)	17(7)
C37	74(15)	71(9)	81(9)	51(7)	24(8)	14(8)
C36	68(12)	68(8)	87(8)	56(6)	29(7)	22(7)
C35	77(9)	73(6)	89(7)	45(5)	23(6)	26(5)
C34	114(10)	81(7)	100(8)	53(6)	13(6)	22(6)
C40	47(7)	29(6)	25(5)	16(5)	19(5)	25(5)
C42	37(3)	35(3)	37(3)	17.3(15)	11.8(13)	12.7(13)
C41	32(7)	62(10)	46(8)	22(7)	-12(6)	-2(7)
C43	180(30)	32(8)	60(11)	20(8)	-16(13)	25(12)
C25	134(11)	182(14)	94(8)	50(9)	35(7)	110(11)
C39	35(3)	34(3)	36(3)	17(2)	12.6(19)	12(2)
O5	91(4)	89(4)	91(4)	41.3(19)	31.4(16)	30.9(16)
C38	71(5)	70(5)	71(5)	32(3)	25(2)	24(2)
C18	73(11)	64(10)	63(9)	32(7)	9(8)	6(8)
C19	132(16)	89(13)	88(11)	21(10)	25(11)	26(12)
C20	151(19)	150(20)	110(16)	23(15)	32(14)	67(16)
C21	220(30)	150(30)	110(20)	10(20)	0(20)	90(30)
C17	140(20)	160(20)	170(30)	100(20)	-29(18)	-46(17)
C16	142(19)	129(14)	150(20)	79(14)	-20(16)	-32(12)
C15	124(15)	133(15)	119(17)	77(14)	10(12)	-21(12)
C14	108(15)	69(12)	92(15)	40(11)	34(12)	13(10)
C13	51(11)	94(16)	106(17)	64(14)	31(11)	13(11)
O3	95(13)	69(11)	86(11)	41(10)	52(10)	24(11)
O2	88(11)	80(10)	101(12)	53(10)	66(10)	45(10)
C45	199(12)	68(5)	133(7)	74(5)	118(8)	83(7)

C46	226(15)	81(6)	176(9)	67(6)	147(10)	83(8)
C47	175(13)	88(7)	154(9)	69(6)	102(9)	74(7)
C48	94(7)	56(5)	136(7)	55(5)	62(6)	43(5)
C49	105(8)	66(5)	122(7)	63(5)	63(6)	42(5)
C44	84(5)	50(4)	105(6)	61(4)	71(5)	45(4)
C0AA	100(20)	85(19)	140(30)	56(19)	80(20)	24(16)

Table S4. Bond Lengths for Cluster 1.

Atom	Atom	Length/Å	Atom	Atom	Length/Å
S4	P3	2.015(3)	Ag4	Ag1A[1]	2.810(5)
S4	Ag6	2.466(2)	Ag4	Ag5	2.824(5)
S4	Ag2[1]	2.449(2)	Ag1	Ag1A	2.3246(17)
S4	Ag1[1]	2.653(2)	Ag1	Ag1A[1]	3.0502(18)
S4	Ag5	2.715(2)	Ag1	Ag5[1]	2.3205(17)
S2	P1	2.005(3)	Ag1	Ag5	3.1105(18)
S2	Ag6	2.540(2)	Ag1A	Ag5[1]	3.0518(17)
S2	Ag4	2.334(5)	Ag1A	Ag5	2.4716(16)
S2	Ag3	2.681(2)	C27	C28	1.385(13)
S2	Ag5	2.533(2)	C27	C26	1.374(14)
P3	S6	2.007(3)	C28	C29	1.374(13)
P3	O7	1.596(6)	C29	C30	1.388(12)
P3	C26	1.810(16)	C30	C31	1.378(12)
P3	C32	1.79(2)	C31	C26	1.384(13)
P2	S3	2.003(3)	C8	C7	1.381(15)
P2	S5	2.019(3)	C8	C9	1.388(14)
P2	O8	1.646(8)	C7	C12	1.381(15)
P2	C44	1.713(8)	C12	C11	1.389(14)
P1	S1	2.019(3)	C11	C10	1.392(15)
P1	O1	1.575(7)	C10	C9	1.394(14)
P1	C7	1.75(2)	C10	O2	1.36(2)
P1	C1	1.837(17)	C3	C4	1.387(14)
S6	Ag7	2.482(3)	C3	C2	1.384(14)
S6	Ag4[1]	2.451(4)	C4	C5	1.386(14)
S6	Ag3[1]	2.655(2)	C4	O3	1.378(10)

S6	Ag1A	2.580(2)	C5	C6	1.381(14)
S3	Ag2	2.565(3)	C6	C1	1.380(14)
S3	Ag4	2.372(5)	C1	C2	1.374(14)
S3	Ag1	2.595(2)	C33	C32	1.383(17)
S3	Ag3	2.579(2)	C33	C34	1.389(16)
S5	Ag6	2.384(3)	C32	C37	1.379(17)
S5	Ag7	2.342(2)	C37	C36	1.395(16)
S5	Ag1A	2.740(2)	C36	C35	1.376(17)
S5	Ag5	2.702(2)	C35	C34	1.390(16)
S1	Ag7[1]	2.436(3)	C35	O5	1.37(2)
S1	Ag2[1]	2.362(3)	C40	C41	1.507(19)
S1	Ag1[1]	2.826(3)	C42	C41	1.53(2)
S1	Ag1A[1]	2.601(3)	C42	C43	1.52(2)
O7	C22	1.452(9)	O5	C38	1.4800(10)
O8	C40	1.398(13)	C18	C19	1.47(2)
O6	C29	1.386(15)	C19	C20	1.48(3)
O6	C39	1.478(16)	C20	C21	1.45(5)
O1	C18	1.35(2)	C21	C21[2]	1.88(10)
O1	C14	1.12(3)	C17	C17[3]	1.52(10)
C22	C23	1.441(13)	C17	C16	1.41(3)
O4	C50	1.394(18)	C16	C15	1.39(3)
O4	C47	1.392(16)	C15	C14	1.41(3)
C24	C23	1.432(16)	C13	O3	1.36(3)
C24	C25	1.49(2)	O2	C0AA	1.45(3)
Ag6	Ag7	3.3166(17)	C45	C46	1.385(11)
Ag6	Ag3	2.9773(15)	C45	C44	1.385(10)
Ag7	Ag2	3.301(2)	C46	C47	1.337(12)
Ag7	Ag3[1]	2.9005(18)	C47	C48	1.326(11)
Ag2	Ag3	2.8890(19)	C48	C49	1.379(11)
Ag4	Ag1	2.761(4)	C49	C44	1.412(9)

[1]-X,-Y,-Z; [2]-X,1-Y,1-Z; [3]1-X,1-Y,1-Z

Table S5. Bond Angles for Cluster 1.

Atom	Atom	Atom	Angle/°	Atom	Atom	Atom	Angle/°
P3	S4	Ag6	107.28(10)	Ag5[1]	Ag1	S3	161.61(8)
P3	S4	Ag2[1]	112.50(10)	Ag5[1]	Ag1	S1[1]	94.17(7)
P3	S4	Ag1[1]	100.50(9)	Ag5[1]	Ag1	Ag4	109.67(11)
P3	S4	Ag5	105.93(10)	Ag5[1]	Ag1	Ag1A[1]	52.69(4)
Ag2[1]	S4	Ag6	89.79(7)	Ag5[1]	Ag1	Ag1A	82.14(6)
Ag1[1]	S4	Ag5	51.21(5)	Ag5[1]	Ag1	Ag5	92.84(6)
P1	S2	Ag6	109.24(12)	S2	Ag3	Ag6	53.02(5)
P1	S2	Ag4	115.07(15)	S2	Ag3	Ag7[1]	94.94(7)
P1	S2	Ag3	107.29(10)	S2	Ag3	Ag2	152.31(7)
P1	S2	Ag5	103.34(11)	S6[1]	Ag3	S2	102.30(7)
Ag6	S2	Ag3	69.48(6)	S6[1]	Ag3	Ag6	151.28(7)
Ag4	S2	Ag5	70.78(12)	S6[1]	Ag3	Ag7[1]	52.84(6)
S6	P3	S4	117.68(11)	S6[1]	Ag3	Ag2	98.58(6)
O7	P3	S4	108.2(2)	S3	Ag3	S2	100.67(8)
O7	P3	S6	108.9(2)	S3	Ag3	S6[1]	106.34(8)
O7	P3	C26	98.5(6)	S3	Ag3	Ag6	93.93(6)
O7	P3	C32	101.3(8)	S3	Ag3	Ag7[1]	156.63(7)
C26	P3	S4	113.3(7)	S3	Ag3	Ag2	55.60(6)
C26	P3	S6	108.4(5)	Ag7[1]	Ag3	Ag6	109.38(5)
C32	P3	S4	102.7(8)	Ag2	Ag3	Ag6	109.73(5)
C32	P3	S6	116.6(7)	S6	Ag1A	S5	101.04(7)
S3	P2	S5	117.63(12)	S6	Ag1A	S1[1]	111.61(8)
O8	P2	S3	110.9(3)	S6	Ag1A	Ag4[1]	53.88(10)
O8	P2	S5	110.0(3)	S6	Ag1A	Ag1[1]	79.83(6)
O8	P2	C44	97.8(4)	S6	Ag1A	Ag5[1]	111.28(7)
C44	P2	S3	110.9(3)	S5	Ag1A	Ag4[1]	149.97(12)
C44	P2	S5	107.7(3)	S5	Ag1A	Ag1[1]	108.03(7)
S2	P1	S1	118.35(13)	S5	Ag1A	Ag5[1]	142.27(7)
O1	P1	S2	108.8(5)	S1[1]	Ag1A	S5	102.47(8)
O1	P1	S1	109.1(4)	S1[1]	Ag1A	Ag4[1]	102.52(11)
O1	P1	C7	102.1(6)	S1[1]	Ag1A	Ag1[1]	144.66(7)
O1	P1	C1	104.2(6)	S1[1]	Ag1A	Ag5[1]	83.65(6)
C7	P1	S2	103.0(6)	Ag4[1]	Ag1A	Ag1[1]	56.05(10)
C7	P1	S1	114.1(5)	Ag4[1]	Ag1A	Ag5[1]	57.42(9)
C1	P1	S2	112.5(6)	Ag1	Ag1A	S6	160.14(8)
C1	P1	S1	102.9(6)	Ag1	Ag1A	S5	97.83(7)
P3	S6	Ag7	110.94(11)	Ag1	Ag1A	S1[1]	69.76(7)

P3	S6	Ag4[1]	114.13(15)	Ag1	Ag1A	Ag4[1]	106.26(10)
P3	S6	Ag3[1]	104.85(9)	Ag1	Ag1A	Ag1[1]	88.54(5)
P3	S6	Ag1A	108.04(10)	Ag1[1]	Ag1A	Ag5[1]	61.29(4)
Ag4[1]	S6	Ag3[1]	23.94(10)	Ag1	Ag1A	Ag5	80.80(6)
Ag4[1]	S6	Ag1A	67.85(12)	Ag1	Ag1A	Ag5[1]	48.87(4)
Ag1A	S6	Ag3[1]	47.69(5)	Ag5	Ag1A	S6	102.63(7)
P2	S3	Ag2	108.13(11)	Ag5	Ag1A	S5	62.20(6)
P2	S3	Ag4	115.01(16)	Ag5	Ag1A	S1[1]	144.87(8)
P2	S3	Ag1	105.87(10)	Ag5	Ag1A	Ag4[1]	103.83(11)
P2	S3	Ag3	107.58(12)	Ag5	Ag1A	Ag1[1]	48.31(4)
Ag2	S3	Ag3	68.35(7)	Ag5	Ag1A	Ag5[1]	91.34(5)
Ag4	S3	Ag1	67.37(12)	S4	Ag5	Ag4	154.12(12)
P2	S5	Ag6	107.91(10)	S4	Ag5	Ag1	140.23(6)
P2	S5	Ag7	111.48(12)	S4	Ag5	Ag1A[1]	108.64(6)
P2	S5	Ag1A	106.17(10)	S2	Ag5	S4	108.39(8)
P2	S5	Ag5	100.71(9)	S2	Ag5	S5	111.33(8)
Ag7	S5	Ag6	89.13(8)	S2	Ag5	Ag4	51.32(11)
Ag5	S5	Ag1A	54.03(5)	S2	Ag5	Ag1	106.52(7)
P1	S1	Ag7[1]	110.46(13)	S2	Ag5	Ag1A[1]	80.66(6)
P1	S1	Ag2[1]	110.29(12)	S5	Ag5	S4	102.17(7)
P1	S1	Ag1[1]	105.18(11)	S5	Ag5	Ag4	100.82(11)
P1	S1	Ag1A[1]	100.72(12)	S5	Ag5	Ag1	82.03(6)
Ag1A[1]	S1	Ag1[1]	50.52(5)	S5	Ag5	Ag1A[1]	141.32(7)
C22	O7	P3	120.3(5)	Ag4	Ag5	Ag1	55.21(10)
C40	O8	P2	125.0(6)	Ag4	Ag5	Ag1A[1]	56.98(9)
C29	O6	C39	114.1(9)	Ag1[1]	Ag5	S4	63.03(6)
C18	O1	P1	137.0(12)	Ag1[1]	Ag5	S2	110.79(8)
C14	O1	P1	136.9(15)	Ag1[1]	Ag5	S5	137.88(8)
C23	C22	O7	109.9(7)	Ag1[1]	Ag5	Ag4	105.94(10)
C47	O4	C50	109.0(14)	Ag1[1]	Ag5	Ag1	87.16(6)
C23	C24	C25	116.2(13)	Ag1[1]	Ag5	Ag1A[1]	48.99(4)
C24	C23	C22	114.2(9)	Ag1[1]	Ag5	Ag1A	79.00(6)
S4	Ag6	S2	116.53(8)	Ag1A	Ag5	S4	98.44(7)
S4	Ag6	Ag7	93.03(6)	Ag1A	Ag5	S2	153.06(9)
S4	Ag6	Ag3	121.10(6)	Ag1A	Ag5	S5	63.77(6)
S2	Ag6	Ag7	132.85(7)	Ag1A	Ag5	Ag4	102.27(11)
S2	Ag6	Ag3	57.49(6)	Ag1A[1]	Ag5	Ag1	59.33(4)
S5	Ag6	S4	120.68(8)	Ag1A	Ag5	Ag1	47.54(4)
S5	Ag6	S2	122.80(9)	Ag1A	Ag5	Ag1A[1]	88.66(5)

S5	Ag6	Ag7	44.92(6)	C26	C27	C28	120.8(14)
S5	Ag6	Ag3	91.44(6)	C29	C28	C27	118.0(13)
Ag3	Ag6	Ag7	76.08(4)	O6	C29	C30	113.5(10)
S6	Ag7	Ag6	89.83(6)	C28	C29	O6	124.3(11)
S6	Ag7	Ag2	137.75(7)	C28	C29	C30	122.1(11)
S6	Ag7	Ag3[1]	58.50(6)	C31	C30	C29	118.9(10)
S5	Ag7	S6	116.71(9)	C30	C31	C26	119.6(11)
S5	Ag7	S1[1]	121.31(10)	C27	C26	P3	121.3(11)
S5	Ag7	Ag6	45.95(6)	C27	C26	C31	120.5(12)
S5	Ag7	Ag2	94.37(7)	C31	C26	P3	118.2(11)
S5	Ag7	Ag3[1]	124.54(8)	C7	C8	C9	121.6(13)
S1[1]	Ag7	S6	121.25(9)	C8	C7	P1	121.2(11)
S1[1]	Ag7	Ag6	138.44(8)	C12	C7	P1	120.2(12)
S1[1]	Ag7	Ag2	45.60(7)	C12	C7	C8	118.2(15)
S1[1]	Ag7	Ag3[1]	93.86(7)	C7	C12	C11	121.7(14)
Ag2	Ag7	Ag6	92.93(5)	C12	C11	C10	119.3(13)
Ag3[1]	Ag7	Ag6	78.94(4)	C11	C10	C9	119.4(12)
Ag3[1]	Ag7	Ag2	80.72(5)	O2	C10	C11	125.1(15)
S4[1]	Ag2	S3	119.39(8)	O2	C10	C9	115.2(15)
S4[1]	Ag2	Ag7	135.48(8)	C8	C9	C10	119.6(12)
S4[1]	Ag2	Ag3	90.30(6)	C2	C3	C4	120.4(13)
S3	Ag2	Ag7	88.85(6)	C5	C4	C3	120.3(12)
S3	Ag2	Ag3	56.05(6)	O3	C4	C3	114.9(14)
S1[1]	Ag2	S4[1]	120.02(9)	O3	C4	C5	124.8(14)
S1[1]	Ag2	S3	120.58(8)	C6	C5	C4	118.0(13)
S1[1]	Ag2	Ag7	47.45(6)	C1	C6	C5	122.2(12)
S1[1]	Ag2	Ag3	124.09(8)	C6	C1	P1	116.0(10)
Ag3	Ag2	Ag7	77.49(5)	C2	C1	P1	124.8(11)
S2	Ag4	S6[1]	120.5(2)	C2	C1	C6	119.3(13)
S2	Ag4	S3	118.71(18)	C1	C2	C3	119.8(12)
S2	Ag4	Ag1	125.57(19)	C32	C33	C34	121.6(16)
S2	Ag4	Ag1A[1]	89.46(17)	C33	C32	P3	120.9(15)
S2	Ag4	Ag5	57.89(12)	C37	C32	P3	121.1(15)
S6[1]	Ag4	Ag1	88.11(14)	C37	C32	C33	117.8(18)
S6[1]	Ag4	Ag1A[1]	58.27(11)	C32	C37	C36	121.7(17)
S6[1]	Ag4	Ag5	123.85(19)	C35	C36	C37	119.5(14)
S3	Ag4	S6[1]	120.6(2)	C36	C35	C34	119.9(14)
S3	Ag4	Ag1	60.17(12)	O5	C35	C36	126.1(16)
S3	Ag4	Ag1A[1]	126.47(18)	O5	C35	C34	114.0(16)

S3	Ag4	Ag5	90.82(14)	C33	C34	C35	119.4(14)
Ag1	Ag4	Ag1A[1]	66.39(10)	O8	C40	C41	106.5(10)
Ag1	Ag4	Ag5	67.68(10)	C43	C42	C41	111.7(12)
Ag1A[1]	Ag4	Ag5	65.60(11)	C40	C41	C42	116.4(11)
S4[1]	Ag1	S1[1]	98.98(7)	C35	O5	C38	115.2(15)
S4[1]	Ag1	Ag4	103.82(10)	O1	C18	C19	116.8(19)
S4[1]	Ag1	Ag1A[1]	86.83(6)	C18	C19	C20	115(3)
S4[1]	Ag1	Ag5	146.07(7)	C21	C20	C19	126(4)
S3	Ag1	S4[1]	111.12(8)	C20	C21	C21[2]	92(4)
S3	Ag1	S1[1]	104.21(8)	C16	C17	C17[3]	126(5)
S3	Ag1	Ag4	52.46(11)	C15	C16	C17	126(4)
S3	Ag1	Ag1A[1]	109.96(7)	C16	C15	C14	122(4)
S3	Ag1	Ag5	80.66(7)	O1	C14	C15	131(2)
S1[1]	Ag1	Ag1A[1]	140.51(7)	C13	O3	C4	115.4(17)
S1[1]	Ag1	Ag5	109.01(7)	C10	O2	C0AA	117.4(19)
Ag4	Ag1	S1[1]	152.12(10)	C46	C45	C44	123.8(9)
Ag4	Ag1	Ag1A[1]	57.57(10)	C47	C46	C45	122.7(11)
Ag4	Ag1	Ag5	57.11(10)	C46	C47	O4	117.2(11)
Ag1A	Ag1	S4[1]	140.63(7)	C48	C47	O4	126.0(10)
Ag1A	Ag1	S3	106.39(6)	C48	C47	C46	116.7(11)
Ag1A	Ag1	S1[1]	59.72(6)	C47	C48	C49	121.3(9)
Ag1A	Ag1	Ag4	108.25(11)	C48	C49	C44	125.1(8)
Ag1A	Ag1	Ag1A[1]	91.46(5)	C45	C44	P2	124.7(5)
Ag1A[1]	Ag1	Ag5	59.38(4)	C45	C44	C49	109.5(7)
Ag1A	Ag1	Ag5	51.66(4)	C49	C44	P2	123.8(6)
Ag5[1]	Ag1	S4[1]	65.77(6)				

1- X,-Y,-Z; [2]-X,1-Y,1-Z; [3]1-X,1-Y,1-Z

Table S6. Torsion Angles for Cluster 1.

A	B	C	D	Angle/°	A	B	C	D	Angle/°
S4	P3	O7	C22	65.3(6)	C30	C31	C26	C27	1(3)
S4	P3	C26	C27	-100.4(17)	C26	P3	O7	C22	-176.6(8)
S4	P3	C26	C31	76.9(18)	C26	C27	C28	C29	0.4(18)
S4	P3	C32	C33	55.3(19)	C8	C7	C12	C11	0.6(19)
S4	P3	C32	C37	-128.5(15)	C7	P1	O1	C18	-132.7(18)
S2	P1	O1	C18	-24.2(18)	C7	C8	C9	C10	1(3)
S2	P1	O1	C14	-144(2)	C7	C12	C11	C10	-4(3)
S2	P1	C7	C8	-91.6(12)	C12	C11	C10	C9	6(3)
S2	P1	C7	C12	95.9(12)	C12	C11	C10	O2	-180.0(19)
S2	P1	C1	C6	40.5(17)	C11	C10	C9	C8	-4(3)
S2	P1	C1	C2	-139.8(17)	C11	C10	O2	C0AA	16(3)
P3	O7	C22	C23	-164.5(7)	C9	C8	C7	P1	-171.8(16)
P3	C32	C37	C36	-178(2)	C9	C8	C7	C12	0.8(17)
P2	O8	C40	C41	172.5(8)	C9	C10	O2	C0AA	-169(2)
P1	O1	C18	C19	129.9(18)	C3	C4	C5	C6	-0.2(16)
P1	O1	C14	C15	129(3)	C3	C4	O3	C13	-176.5(18)
P1	C7	C12	C11	173.3(19)	C4	C3	C2	C1	1(3)
P1	C1	C2	C3	178.8(15)	C4	C5	C6	C1	-1(2)
S6	P3	O7	C22	-63.7(6)	C5	C4	O3	C13	6(3)
S6	P3	C26	C27	32.1(19)	C5	C6	C1	P1	-178.8(12)
S6	P3	C26	C31	-150.5(15)	C5	C6	C1	C2	1(3)
S6	P3	C32	C33	-174.6(15)	C6	C1	C2	C3	-2(3)
S6	P3	C32	C37	1.6(18)	C1	P1	O1	C14	95(2)
S3	P2	O8	C40	-49.4(9)	C2	C3	C4	C5	0.2(18)
S3	P2	C44	C45	40.8(11)	C2	C3	C4	O3	-177.8(19)
S3	P2	C44	C49	-156.8(8)	C33	C32	C37	C36	-2.1(19)
S5	P2	O8	C40	82.5(9)	C32	P3	O7	C22	172.9(9)
S5	P2	C44	C45	-89.2(10)	C32	C33	C34	C35	3(4)
S5	P2	C44	C49	73.2(9)	C32	C37	C36	C35	4(3)
S1	P1	O1	C18	106.3(17)	C37	C36	C35	C34	-2(4)
S1	P1	O1	C14	-14(2)	C37	C36	C35	O5	-180(2)
S1	P1	C7	C8	138.7(11)	C36	C35	C34	C33	-2(4)
S1	P1	C7	C12	-33.7(13)	C36	C35	O5	C38	-3(4)
S1	P1	C1	C6	-88.0(16)	C34	C33	C32	P3	175(2)
S1	P1	C1	C2	91.7(18)	C34	C33	C32	C37	-2(2)
O7	P3	C26	C27	145.4(17)	C34	C35	O5	C38	179(2)
O7	P3	C26	C31	-37.2(18)	C43	C42	C41	C40	177.5(17)

O7	P3	C32	C33	-56.6(19)	C25	C24	C23	C22	57.6(14)
O7	P3	C32	C37	119.6(15)	C39	O6	C29	C28	1.2(18)
O7	C22	C23	C24	60.7(12)	C39	O6	C29	C30	-179.6(12)
O8	P2	C44	C45	156.8(10)	O5	C35	C34	C33	176(2)
O8	P2	C44	C49	-40.8(10)	C18	C19	C20	C21	-61(5)
O8	C40	C41	C42	47.5(17)	C19	C20	C21	C21[1]	-178(4)
O6	C29	C30	C31	-179.6(14)	C17[2]	C17	C16	C15	45(7)
O1	P1	C7	C8	21.2(14)	C17	C16	C15	C14	-173(3)
O1	P1	C7	C12	-151.2(12)	C16	C15	C14	O1	38(5)
O1	P1	C1	C6	158.2(15)	O3	C4	C5	C6	177.5(18)
O1	P1	C1	C2	-22(2)	O2	C10	C9	C8	-179.2(18)
O1	C18	C19	C20	-63(4)	C45	C46	C47	O4	-180.0(17)
O4	C47	C48	C49	-178.1(16)	C45	C46	C47	C48	-1.1(16)
C50	O4	C47	C46	-172.2(17)	C46	C45	C44	P2	175.6(10)
C50	O4	C47	C48	9(3)	C46	C45	C44	C49	11.0(16)
C27	C28	C29	O6	179.5(15)	C46	C47	C48	C49	3(2)
C27	C28	C29	C30	0.3(16)	C47	C48	C49	C44	2(2)
C28	C27	C26	P3	176.3(15)	C48	C49	C44	P2	-173.8(10)
C28	C27	C26	C31	-1(3)	C48	C49	C44	C45	-9.1(16)
C28	C29	C30	C31	0(2)	C44	P2	O8	C40	-165.3(9)
C29	C30	C31	C26	0(3)	C44	C45	C46	C47	-6.9(16)
C30	C31	C26	P3	-176.4(14)					

[1]-X,1-Y,1-Z; [2]1-X,1-Y,1-Z

Table S7. Hydrogen Atom Coordinates (Å×10[4]) and Isotropic Displacement Parameters (Å[2]×10[3]) for Cluster 1.

Atom	*x*	*y*	*z*	U(eq)
H8	-1601.17	2624.56	-1669.12	122
H22A	4406.31	928.24	1609.2	71
H22B	4728.95	640.66	606.88	71
H24A	6596.58	2916.72	2967.45	123
H24B	7360.63	2205.49	3244.86	123
H23A	6172.54	626.92	1828.28	91
H23B	6678.9	1629.87	1564.06	91
H50A	-2216.03	7364.35	2276.76	293
H50B	-811.48	7781.27	2744.53	293
H50C	-1540.68	8200.45	3453.46	293
H27	2821.19	3629.7	-32.74	58
H28	3446.56	5501.56	199.4	58
H30	6286.64	6257.73	2599.59	55
H31	5646.9	4384.76	2353.57	53
H8A	-151.09	2585.93	4976.53	74
H12	-1685.38	-586.7	2734.32	75
H11	-2993.9	-1011.98	3518.13	95
H9	-1391.93	2169.82	5814.95	88
H3	-1037.04	606.21	5784.49	91
H5	-3572.65	-589.02	3053.3	65
H6	-2138.63	106.58	2484.29	53
H2	377.39	1289.18	5189.85	80
H33	5005.87	4478.17	2834.61	105
H37	3326.41	3738.07	-43.96	87
H36	3894.9	5666.98	373.87	81
H34	5753.49	6390.02	3229.97	122
H40A	-2709.53	1500.41	-2391.26	35
H40B	-1417.49	2092.13	-2355.1	35
H42A	-1869.36	4487.16	-1784.28	43
H42B	-1111.26	4096.38	-2474.09	43
H41A	-3422.85	2736.17	-2805.43	67
H41B	-2708.93	2347.13	-3532.33	67
H43A	-3124.12	3855.38	-3767.36	155
H43B	-1891.75	4840.46	-3386.62	155
H43C	-2799.46	5039.59	-2809.83	155
H25A	5543.13	1090.16	3300.43	197

H25B	5158.31	2165.22	3404.48	197
H25C	6313.42	2302.81	4236.31	197
H39A	4538.72	6995.95	255.46	52
H39B	5115.26	8282.64	1108.98	52
H39C	3932.48	7465.68	1060.63	52
H38A	5205.42	7388.8	843.8	106
H38B	5367.75	8585.31	1714.32	106
H38C	4117.98	7584.19	1179.4	106
H18A	1815.47	3472.17	3804.14	86
H18B	2863.62	3215.01	4402.27	86
H19A	2872.82	4923.81	5407.82	137
H19B	2520.71	4080.62	5878.8	137
H20A	597.91	3875.02	5283.67	180
H20B	1391.26	5154.33	6023.09	180
H21A	148.65	4302.99	3948.28	220
H21B	1000.62	5613.65	4684.81	220
H16A	3327.58	4602.46	5738.63	196
H16B	2936.58	4280.77	4573.74	196
H15A	3859.19	3135.87	5497.82	165
H15B	3631.83	2837.82	4345.47	165
H14A	2286.9	1616.42	4309.08	110
H14B	2132.75	2448.04	5278.97	110
H13A	-4353.39	-1580.24	3678.53	118
H13B	-4698.5	-480.11	4142.97	118
H13C	-4753.55	-1335.23	4637.86	118
H45	-1750.48	3161.6	1624.88	127
H46	-1930.87	4677.65	2827.71	165
H48	-1069.4	6553.79	1498.71	101
H49	-995.63	5029.81	232.18	101
H0AA	-4279.98	-1103.51	4370.29	152
H0AB	-4034.44	-807.28	5532.85	152
H0AC	-3168.02	-1266.65	4999.07	152

Table S8. EDX elemental constituent of AgNPs

**AgNPs**	**Wt%**	**Wt% sigma**
C	22.91	0.43
O	13.00	0.41
Na	3.53	0.11
P	2.42	0.09
S	6.01	0.11
Ag	52.14	0.42
Total	100.00	
